# Bacteroidetocins Target the Essential Outer Membrane Protein BamA of *Bacteroidales* Symbionts and Pathogens

**DOI:** 10.1128/mBio.02285-21

**Published:** 2021-09-14

**Authors:** Leigh M. Matano, Michael J. Coyne, Leonor García-Bayona, Laurie E. Comstock

**Affiliations:** a Division of Infectious Diseases, Brigham and Women’s Hospital, Harvard Medical School, Boston, Massachusetts, USA; University of Georgia

**Keywords:** *Bacteroides*, BamA, bacteriocin, microbiota

## Abstract

Bacteroidetocins are a family of antibacterial peptide toxins that are produced by and target members of the phylum *Bacteroidetes*. To date, 19 bacteroidetocins have been identified, and four have been tested and shown to kill diverse *Bacteroidales* species (M. J. Coyne, N. Béchon, L. M. Matano, V. L. McEneany, et al., Nat Commun 10:3460, 2019, https://doi.org/10.1038/s41467-019-11494-1). Here, we identify the target and likely mechanism of action of the bacteroidetocins. We selected seven spontaneous mutants of four different genera, all resistant to bacteroidetocin A (Bd-A) and found that all contained mutations in a single gene, *bamA*. Construction of three of these *bamA* mutants in the wild-type (WT) strains confirmed they confer resistance to Bd-A as well as to other bacteroidetocins. We identified an aspartate residue of BamA at the beginning of exterior loop 3 (eL3) that, when altered, renders strains resistant to Bd-A. Analysis of a panel of diverse *Bacteroidales* strains showed a correlation between the presence of this aspartate residue and Bd-A sensitivity. Fluorescence microscopy and transmission electron microscopy (TEM) analysis of Bd-A-treated cells showed cellular morphological changes consistent with a BamA defect. Transcriptomic analysis of Bd-A-treated cells revealed gene expression changes indicative of cell envelope stress. Studies in mice revealed that bacteroidetocin-resistant mutants are outcompeted by their WT strain *in vivo*. Analyses of longitudinal human gut isolates showed that *bamA* mutations leading to bacteroidetocin resistance do not become fixed in the human gut, even in bacteroidetocin-producing strains and nonproducing coresident strains. Together, these data lend further support to the applicability of the bacteroidetocins as therapeutic peptides in the treatment of maladies involving *Bacteroidales* species.

## INTRODUCTION

Studies over the last several years have identified new antibacterial toxins produced by the gut *Bacteroidales*, providing these strains with a competitive advantage in the dense gut ecosystem. These toxins include those delivered by contact-dependent type VI secretion systems (reviewed in reference 1) and secreted diffusible peptides and proteins. Among the diffusible peptides and proteins, three classes have been identified; the membrane attack complex/perforin (MACPF) domain toxins (*Bacteroidales*-secreted antimicrobial proteins [BSAPs]) (2–5), the ubiquitin-like toxin Bacteroides fragilis Ubb (BfUbb) (6), and the bacteroidetocins (7). MACPF domain toxins are produced by diverse members of the phylum *Bacteroidetes* and target the glycan of lipopolysaccharides (LPS) or outer membrane proteins (OMPs) and likely form large pores in the outer membrane (OM). MACPF toxins described to date are specific for intraspecies killing. The BfUbb toxin is produced by a subset of Bacteroides fragilis strains, is 72 amino acids in its mature form, and is 82% similar to human ubiquitin. BfUbb specifically targets a subset of B. fragilis strains.

The bacteroidetocins are ribosomally produced peptide toxins with similarities to class IIa bacteriocins produced by Gram-positive bacteria (7). These molecules are synthesized with a leader sequence that is cleaved at a GG site, yielding mature peptides of 42 to 53 residues. To date, we have identified 19 distinct bacteroidetocins that are produced by diverse members of the *Bacteroidetes* phylum, four of which have been tested and shown to have toxin activity. Unlike the MACPF toxins and BfUbb, the bacteroidetocins have a broader targeting range. Bacteroidetocins A, B, and D (Bd-A, Bd-B, and Bd-D, respectively) kill *Bacteroides*, *Parabacteroides*, and *Prevotella* species (7), which are members of three distinct *Bacteroidales* families. Although these bacteroidetocins target similar strains, their killing profiles are not identical. In addition, not every *Bacteroidales* species is targeted by these bacteroidetocins, and phylogeny alone does not predict which will be sensitive or resistant. Bd-C is less similar to Bd-A, -B, and -D and targets a different set of *Bacteroidales* strains/species (7).

Class IIa bacteriocins target the cytoplasmic membrane components of the mannose phosphotransferase system (PTS) of Gram-positive bacteria, leading to permeabilization of the membrane and loss of cytoplasmic ATP (8–11). *Bacteroidales* lack this PTS, and, unlike the class IIa bacteriocins, bacteroidetocins target Gram-negative *Bacteroidetes*. This study was designed to identify the target of the bacteroidetocins to better understand the variable susceptibility of *Bacteroidales* species to these toxins. In addition, as the bacteroidetocins target not only symbiotic gut species but also pathogenic oral and vaginal *Prevotella* species, a better understanding of how these toxic peptides kill cells is important to evaluate their utility in the treatment of maladies such as periodontal disease and bacterial vaginosis involving members of this order.

## RESULTS

### Selection of Bd-A resistant mutants.

**FIG 1:**
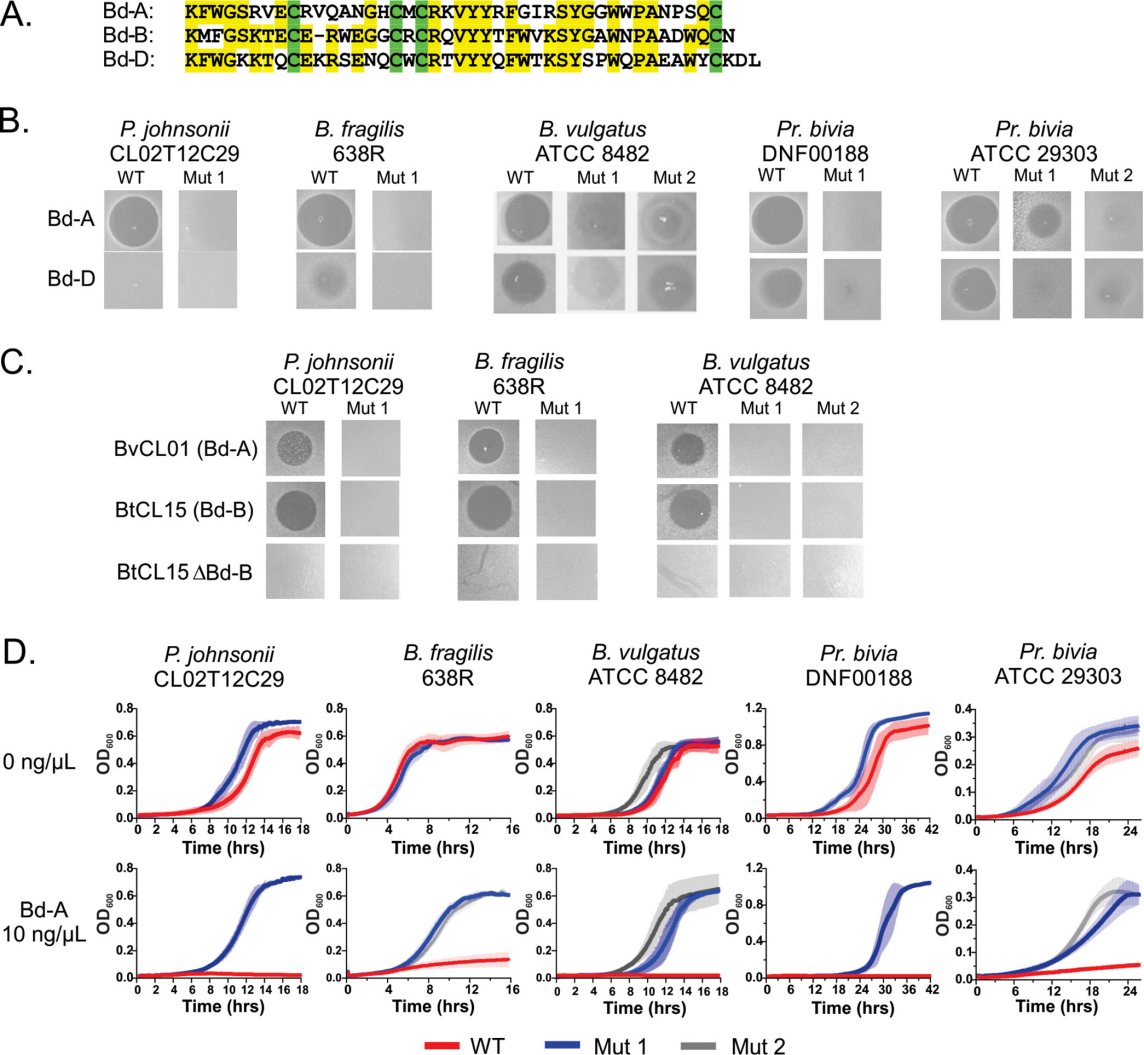
Identification of spontaneous mutants resistant to Bd-A. (A) Sequences of the mature forms of Bd-A and Bd-D. Conserved cysteine residues are highlighted in green, and identical residues are highlighted in yellow. (B) Agar spot overlay assays showing seven bacteroidetocin-resistant mutants selected from five different *Bacteroidales* strains. Mutants selected for resistance to Bd-A were cross resistant to Bd-D (with the exception of *P. johnsonii* CL02T12C29, which is not sensitive to Bd-D). (C) Overlays of Parabacteroides johnsonii CL02T12C29, Bacteroides fragilis 638R, Bacteroides vulgatus ATCC 8482, and *bamA* mutants. Spots are Bd-A-producing Bacteroides vulgatus CL01T12C17 (BvCL01) and Bd-B-producing Bacteroides thetaiotaomicron CL15T12C11 (BtCL15). BtCL15 ΔBd-B lacks the Bd-B-encoding gene and is shown as a control. (D) Growth curves of WT strains and resistant mutants with or without the addition of 10 ng/μl Bd-A. Data were recorded every 10 min and plotted as the averages from at least three biological replicates ± SDs.

The receptors of the MACPF toxins are important fitness determinants for gut colonization but are not essential molecules *in vitro* (3). Therefore, it was possible to identify these receptors using transposon mutagenesis. Attempts to identify *Bacteroides* mutants resistant to Bd-A by transposon mutagenesis were not successful despite repeated attempts, suggesting that the target of Bd-A may be essential. We tested if we could isolate spontaneous mutants resistant to Bd-A by selecting for survivors that continue to grow after repeated passage in the presence of the peptide. For these assays, we used five different wild-type (WT) *Bacteroidales* strains from three different families: Parabacteroides johnsonii CL02T12C29, Bacteroides fragilis 638R, Bacteroides vulgatus ATCC 8482 (also known as Phocaeicola vulgatus), Prevotella bivia DNF00188, and *P. bivia* ATCC 29303. These strains were passaged sequentially in increasing concentrations of Bd-A (Fig. 1A), and those that grew in the presence of the toxin were single-cell cloned and tested individually in overlay assays. With the exception of *P. johnsonii* CL02T12C29, for which the WT strain is not targeted by Bd-D, most strains that grew in the presence of 10 ng/μl Bd-A were resistant to both Bd-A and Bd-D (Fig. 1B). *P. bivia* ATCC 29303 mutant (mut 1) was somewhat resistant to Bd-A (Fig. 1B and D), enough to identify the mutant during selection, but was completely resistant to killing by Bd-D (Fig. 1B). Synthetic Bd-B peptide is insoluble in water at neutral pH; therefore, we tested the resistance of four of these mutant strains to Bd-A and Bd-B produced from native Bd-B-producing *Bacteroides* strains. We found that these mutants were also resistant to these natively produced toxins (Fig. 1C), showing that most Bd-A-resistant mutants are also resistant to Bd-B and Bd-D. Bd-A-resistant mutants were not growth defective *in vitro* and grew well in the presence of 10 ng/μl of Bd-A, a concentration that completely inhibited the growth of the WT strains (Fig. 1D).

### Identification of mutations leading to Bd-A resistance.

To determine what mutations occurred to confer resistance to Bd-A, whole-genome sequencing (WGS) was performed on the five WT strains and the seven resistant mutants. Comparison of WT and mutant genomes revealed that each mutant has an alteration in *bamA* (see Table S1 in the supplemental material), encoding an essential OMP protein of Gram-negative bacteria. BamA is part of the BAM complex responsible for shuttling, assembling, and inserting β-barrel proteins into the OM (reviewed in reference 12). The BAM complex consists of BamA and four other lipoproteins, BamBCDE, of which only BamA and BamD are essential.

10.1128/mBio.02285-21.1 TABLE S1 Strains and primers used in this study. Download Table S1, XLSX file, 0.01 MB. Copyright © 2021 Matano et al. 2021 Matano et al. https://creativecommons.org/licenses/by/4.0/ This content is distributed under the terms of the Creative Commons Attribution 4.0 International license.

**FIG 2:**
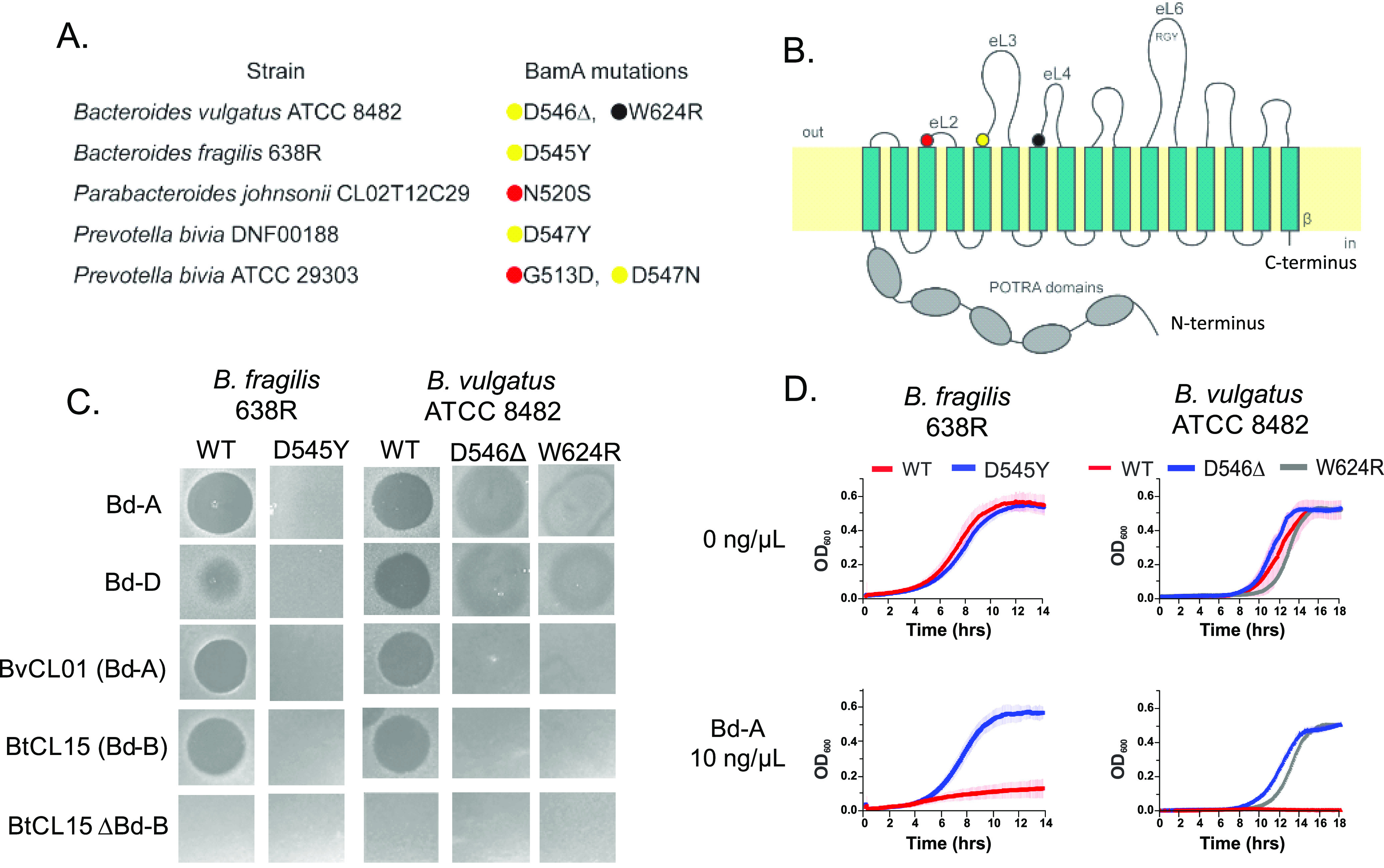
Mutations in specific regions of *bamA* confer resistance to bacteroidetocins. (A) Residues of BamA that are altered in the resistant mutants. (B) Predicted topology of the BamA of B. vulgatus ATCC 8482 and locations of altered residues in Bd-A-resistant mutants mapped to this BamA sequence. Mutations were present in three regions shown here as red/yellow/black spots. All three locations are predicted to be at the N-terminal portion of the extracellular loops (eLs). (C) Several *bamA* mutations were recreated in clean backgrounds of B. fragilis 638R and B. vulgatus ATCC 8482 and they conferred resistance to Bd-A, Bd-D, natively produced Bd-A from BvCL01T12C17 (abbreviated as BvCL01) and natively produced Bd-B from BtCL15T12C11 (abbreviated as BtCL15). Strain BtCL15 ΔBd-B lacks the Bd-B toxin gene and serves as a control. (D) Constructed *bamA* site mutants grew well in liquid culture in the absence and presence of Bd-A at 10 ng/μl.

The mutations identified in the resistant mutants mapped to four residues predicted to be part of extracellular loops 2, 3, or 4 (eL2, eL3, or eL4, respectively) (Fig. 2A and B; see also Fig. S1a and b). These three residues of BamA are each conserved in all four species used for mutation (Fig. S1b). In four of the seven mutants, the aspartate at the beginning of eL3 was altered. Aspartate is negatively charged at physiological pH, and these substitutions removed that charge by several different mutations: a three-base-pair codon deletion (D546Δ) resulting in loss of the aspartate, a mutation resulting in its replacement with the polar but uncharged residue asparagine (D547N), or a mutation changing it to the nonpolar residue tyrosine (D545Y and D547Y) (Fig. 2A and B). The structure of B. vulgatus BamA was modeled with Phyre2 (13) using the crystal structure of the BamA of Neisseria gonorrhoeae (14) (PDB accession 4K3B) (see Fig. S2). In this structural prediction, the residues identified as conferring resistance to Bd-A appear spatially aligned (Fig. S2), potentially allowing for interaction of Bd-A with all three residues. To confirm that these mutations confer resistance, three of these mutations were created in the WT strains B. fragilis 638R (D545Y) and B. vulgatus ATCC 8482 (D546Δ and W624R). As expected, these constructed mutants conferred resistance to the synthesized Bd-A and Bd-D peptides as well as to the native Bd-A and Bd-B toxins produced by BvCL01T12C17 and BtCL15T12C11 (Fig. 2C). These mutants also grew well *in vitro* and were resistant to the bacteroidetocins in liquid culture, similarly to the spontaneous mutants (Fig. 2D).

10.1128/mBio.02285-21.3 FIG S1 Alignment of B. vulgatus 8482 BamA with BamA sequences of other bacteria. (a) Phyre2 alignment of BamA (BVU_0867) to PDB sequences of N. gonorrhoeae (43kbA) and E. coli K-12 (5ekqA). Predicted secondary structure from the three-dimensional (3D) model of BVU_0867 is displayed above the sequence. Percent identity is 25% for BamA of N. gonorrhoeae and 24% for E. coli. The 16 predicted β-strands of the β-barrel are numbered, and eL3 is noted with a blue bar. Loop eL3 is longer in B. vulgatus (Bv) BamA than in the other two species. The site of eL3D is noted by a blue arrowhead below the sequences, and the four starred residues indicate the conserved (V/I)RG(F/Y) motif. (b) Alignment of Bv BamA (BVU_0867) to B. fragilis 638R (BF638R_0513), *P. bivia* DNF00188 (HMPREF1651_07805), *P. johnsonii* CL02T12C29 (HMPREF1077_00359), and P. gingivalis W83 (PG0191). The predicted secondary structure from the 3D model of BVU_0867 is displayed above the sequence. Conserved residues are highlighted in red, similar residues are highlighted in yellow, and the four starred residues indicate the conserved (V/I)RG(F/Y) motif. The 16 predicted β-strands of the β-barrel are numbered and eL3 is noted with a blue bar. The site of eL3D is noted by a blue arrowhead below the sequences; other mutation sites are noted with cyan arrowhead. Illustration was generated using ESPript 3.0. Horizontal arrows indicate β-strands and horizontal helical symbols represent α-helices. Download FIG S1, PDF file, 0.9 MB. Copyright © 2021 Matano et al. 2021 Matano et al. https://creativecommons.org/licenses/by/4.0/ This content is distributed under the terms of the Creative Commons Attribution 4.0 International license.

10.1128/mBio.02285-21.4 FIG S2 Mapping Bd-A-resistant mutant sites onto a predicted BamA structure of B. vulgatus ATCC 8482. (A) BamA structure predicted using Phyre2 and mapping to the crystal structure of Neisseria gonorrhoeae FA 1090 (4K3B). Periplasmic region including the POTRA domains is shown in gray, β-barrel domain is shown in blue. (B) Close up of β-barrel domain. The sites of two Bv mutations (W624R and D546Δ) as well as the equivalent location of the *P. johnsonii* N520S mutation in the C-terminal portion are shown in red. Download FIG S2, PDF file, 0.3 MB. Copyright © 2021 Matano et al. 2021 Matano et al. https://creativecommons.org/licenses/by/4.0/ This content is distributed under the terms of the Creative Commons Attribution 4.0 International license.

### Importance of the aspartate of eL3 for Bd-A sensitivity.

**FIG 3:**
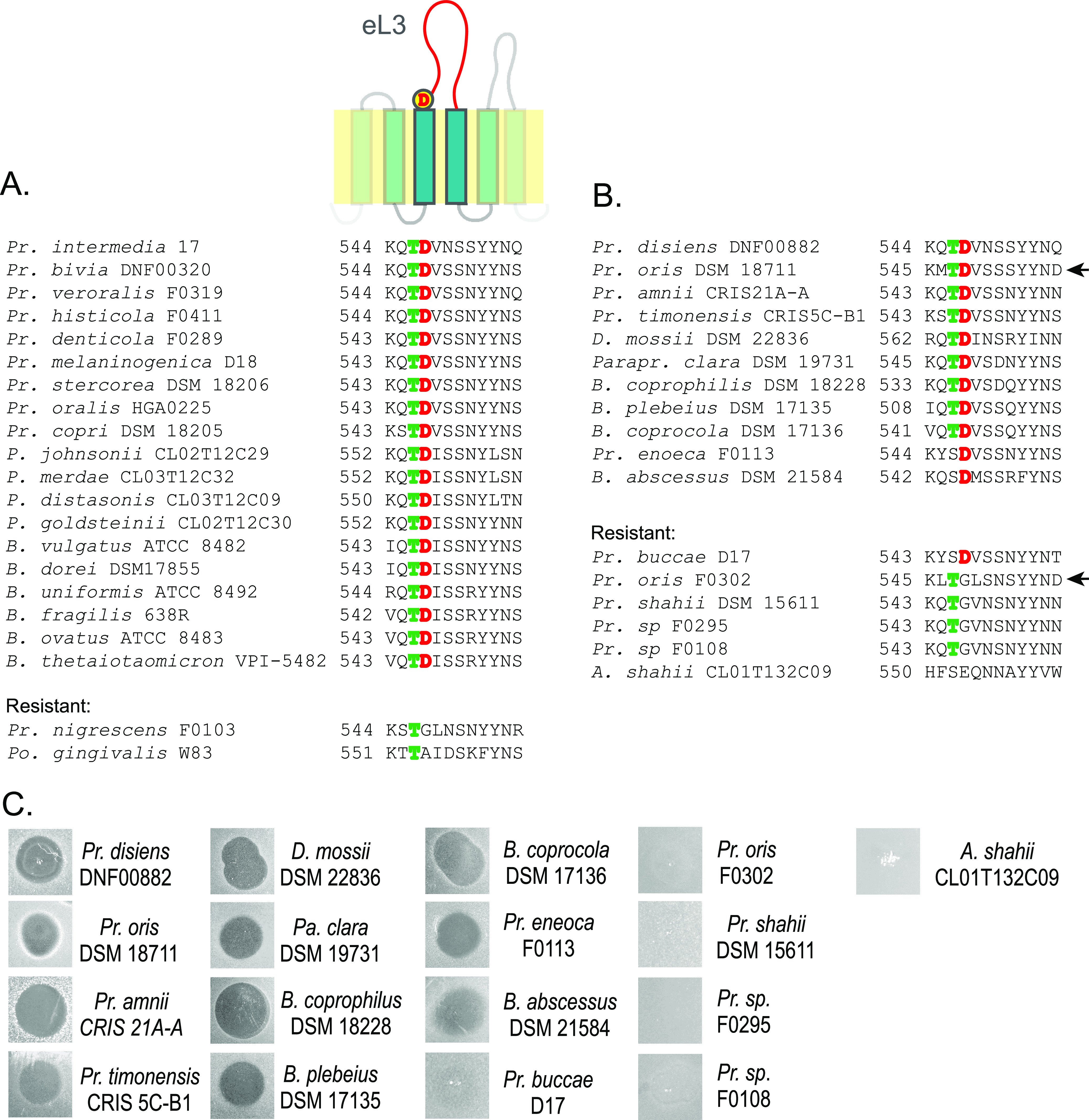
An aspartate residue in extracellular loop 3 (eL3) of BamA correlates with Bd-A sensitivity. (A) Sequence alignment of the BamA sequences in the eL3D region of 21 *Bacteroidales* strains previously tested with Bd-A (7). All resistant strains lack eL3D. (B) Sequence alignments of the eL3 residues of BamA in the eL3D regions of newly tested *Bacteroidales* strains. Arrows point out two *P. oris* strains with different eL3D sequences. (C) Overlays of the additional strains tested for Bd-A sensitivity or resistance. All strains containing eL3D were sensitive, except for P. buccae D17. The two *P. oris* strains have different sensitivities to Bd-A. Genus abbreviations: *Pr*, *Prevotella*; *P*, *Parabacteroides*; *B*, *Bacteroides*; *D*, *Dysgonomonas*; *Parapr*, *Paraprevotella*; *A*, *Alistipes*; *Po*, *Porphyromonas*. Accession numbers for the genomes sequences of each WT strain are provided in Table S1 in the supplemental material.

The mutations affecting the aspartate residue at the beginning of eL3 suggested a charge interaction that may be important for the activity of the bacteroidetocins. To determine the importance of the aspartate residue, we aligned the BamA sequences of all *Bacteroidales* species previously tested for sensitivity to Bd-A (7) and found that all Bd-A-sensitive strains have a BamA with the aspartate residue at the N-terminal end of eL3, whereas the two resistant strains, Prevotella nigrescens F0103 and Porphyromonas gingivalis W83, do not (Fig. 3A). To further correlate this aspartate residue with sensitivity or resistance to Bd-A, we analyzed the BamA sequences of 17 additional *Bacteroidales* strains in our collection or available from public repositories. The new panel of strains included members of the genera *Prevotella*, *Bacteroides* (several species also known as *Phocaeicola*), *Paraprevotella*, *Dysgonomonas*, and *Alistipes* (Fig. 3B). Twelve of these 17 strains contain the aspartate residue at the beginning of eL3 and five do not. We tested each of these 17 strains for Bd-A sensitivity/resistance and found that all five of the strains that lack the aspartate residue at this site are resistant to Bd-A (Fig. 3C). Eleven of the 12 strains that contained eL3D are sensitive to Bd-A, with Prevotella buccae D17 being the only exception. BamA sequences vary between members of the same genera and even members of the same species. Of note are two Prevotella oris strains whose BamA sequences are extremely similar (99.2%), with only seven different residues along the entire 877-residue proteins (see Fig. S3). Four of these differences occur in the region of eL3D, where BamA of *P. oris* F0302 contains a glycine instead of an aspartate (Fig. 3B; Fig. S3). We tested these two strains and found that *P. oris* F0302 is resistant to Bd-A, while *P. oris* DSM 18711 is sensitive (Fig. 3C). These data confirm the importance of eL3D to the activity of Bd-A.

10.1128/mBio.02285-21.5 FIG S3 Comparison of the BamA sequences of two Prevotella oris strains that differ in Bd-A sensitivity. Alignment of BamA sequences of *P. oris* DSM 18711 and *P. oris* F0302. The sequences are nearly identical except for the seven amino acids highlighted in yellow. Critically, DSM 18711 has eL3D (shown in red at position 548), but F0302 has a G at this site, and three other resides in this region are different. The two residues (N513 and W628) shown in other strains to confer resistance to Bd-A are highlighted in turquoise and are identical between the BamAs of the two strains. Download FIG S3, PDF file, 0.06 MB. Copyright © 2021 Matano et al. 2021 Matano et al. https://creativecommons.org/licenses/by/4.0/ This content is distributed under the terms of the Creative Commons Attribution 4.0 International license.

### Microscopic analyses of Bd-A-treated strains.

**FIG 4:**
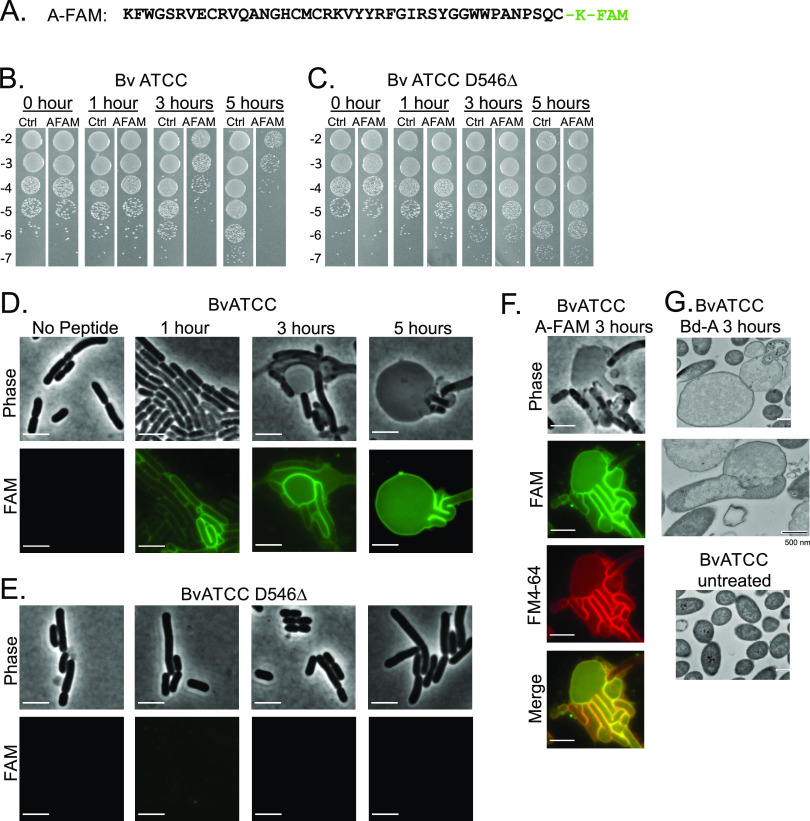
Bd-A localizes to the cell membrane and leads to altered cellular morphology. (A) Sequence of the mature form of Bd-A with lysine-FAM modification at the C-terminus (A-FAM). Time course of A-FAM-treated (10 ng/μl) B. vulgatus ATCC 8482 (B) and its BamA^D546Δ^ mutant (C) over 5 h showing significant loss of CFU of the WT strain but not the mutant over time. Spot dilutions are 10-fold starting with 10^−2^. (D) Fluorescence microscopy of B. vulgatus ATCC 8482 WT treated with A-FAM over 5 h. Over time, cells show abnormal rounding and enlarged size compared to the untreated control. No peptide control are cells grown for 1 h; see Fig. S4 for controls at other time points. (E) Fluorescence microscopy of B. vulgatus ATCC 8482 BamA^D546Δ^ mutant with A-FAM. Images show that cells maintain normal shape upon treatment and have less peptide bound to the surface. Bars, 5 μm. (f) Imaging with fluorescent membrane dye FM4-64 shows Bd-A-FAM localizes to B. vulgatus cell membranes. B. vulgatus WT samples were treated with Bd-A-FAM for 3 h. Merge shows overlap between these two fluorophores. Bars, 5 μm. (G) TEM images of WT B. vulgatus ATCC 8482 treated (top) or untreated (bottom) with Bd-A after 3 h. Some treated cells were enlarged and rounded. Parts of the outer membrane appear to be disrupted or missing. Bars, 500 nm. Additional TEM images at low and high magnification are provided in Fig. S5.

To determine if Bd-A binds the surfaces of sensitive cells but not those of resistant mutants, Bd-A was synthesized with a lysine-carboxyfluorescein (FAM) modification at the C terminus (Bd-A-FAM) (Fig. 4A). We first confirmed that the fluorescently labeled peptide is still toxic to WT but not to mutant cells. We chose to analyze WT B. vulgatus ATCC 8482 and both of the BamA mutants of this strain: D546Δ and W624R. We found that the CFU count of the WT strain treated with 10 ng/μl Bd-A-FAM decreased within 3 h, with an approximately 3-log decrease by 5 h (Fig. 4B). In contrast, the CFU count of the two *bamA* mutants treated with Bd-A-FAM increased over time equivalent to the untreated control (Fig. 4C [D546Δ] and Fig. S4B [W624R]). These data show that the fluorescently labeled Bd-A remains toxic to the WT but does not affect the *bamA* mutants. Next, WT B. vulgatus and the two *bamA* mutants were incubated with Bd-A-FAM for 1, 3, and 5 h and then observed for surface labeling and cellular morphology (Fig. 4D and E; Fig. S4). WT cells treated with Bd-A-FAM showed bright green fluorescence on the cell surface that was nearly undetectable in both *bamA* mutants (Fig. 4E; Fig. S4), further supporting a direct interaction of Bd-A with BamA. Bd-A-FAM colocalized with the membrane staining fluorophore FM4-64 (Fig. 4F). At 3 h, the morphology of the WT strain showed cell rounding that was not seen in the mutant strains (Fig. 4F and G; Fig. S4 and S5). By 5 h, the WT cells displayed significant cell rounding and large membrane protrusions, whereas the mutant strains remained unchanged. WT B. vulgatus ATCC 8482 cells treated with Bd-A for 3 h were fixed, embedded, and sectioned for transmission electron microscopy (TEM) imaging. Cross-sectional analyses showed severe OM defects, including ruptures and ballooning of the OM, which were not evident in the untreated control (Fig. 4G; Fig. S5). These phenotypes have been described in several bacteria when BamA activity is inhibited either with a toxin (15), with BamA inhibitors (16), or when knocked down with CRISPR interference (CRISPRi) (17). These data further support that Bd-A interacts with BamA and likely inhibits its function.

10.1128/mBio.02285-21.6 FIG S4 Bd-A-FAM treatment of B. vulgatus ATCC 8482 BamA^W624R^ and Bd-A-FAM control images. (A) Overlays of B. vulgatus ATCC 8482 BamA^W624R^ show that Bd-A-FAM does not inhibit growth. (B) B. vulgatus ATCC 8482 BamA^W624R^ treated with 10 ng/μl of Bd-A-FAM continues to grow over time, similarly to the untreated control. (C) Fluorescence microscopy of the B. vulgatus ATCC 8482 BamA^W624R^ mutant with A-FAM. Images show that cells maintain normal shape upon treatment and little association of Bd-A-FAM with cells. Bars, 5 μm. (D) Untreated B. vulgatus ATCC 8482 wild-type (WT) at 3 and 5 h. These photos are controls for the 3- and 5-h time points shown in Fig. 4 in the main text. Bars, 5 μm. (E) Uncropped images of B. vulgatus WT untreated and treated with 10 ng/μl of Bd-A for 5 h. The treated sample shows many rounded cells brightly fluorescing. Bars, 20 μm. (F) Untreated B. vulgatus ATCC 8482 BamA^D546Δ^ mutant cells at 3 and 5 h. These photos are controls for the 3- and 5-h time points shown in Fig. 4 in the main text. Bars, 5 μm. (G) Uncropped images of B. vulgatus BamA^D546Δ^ mutant untreated and treated with 10 ng/μl of Bd-A-FAM. Images show that the mutant strain does not have visibly bound Bd-A-FAM, unlike the treated wild-type sample. Bars, 20 μm. Download FIG S4, PDF file, 0.2 MB. Copyright © 2021 Matano et al. 2021 Matano et al. https://creativecommons.org/licenses/by/4.0/ This content is distributed under the terms of the Creative Commons Attribution 4.0 International license.

10.1128/mBio.02285-21.7 FIG S5 TEM imaging of B. vulgatus ATCC 8482 treated with Bd-A shows rounding and outer membrane defects. (A) Cross section of fixed B. vulgatus ATCC 8482 WT cells. (B) Close up of healthy WT cells. (C) Cross section of fixed B. vulgatus ATCC 8482 treated with Bd-A. (D to E) Higher magnification of large abnormal cells. Cells show breaks and loss of outer cell membrane (white arrowheads). Cells also show abnormal accumulation of debris between inner and outer membranes (red arrowheads). Download FIG S5, PDF file, 0.4 MB. Copyright © 2021 Matano et al. 2021 Matano et al. https://creativecommons.org/licenses/by/4.0/ This content is distributed under the terms of the Creative Commons Attribution 4.0 International license.

### Exposure of sensitive cells to Bd-A results in transcriptional changes indicative of cell envelope stress.

To provide further evidence that Bd-A interferes with the function of BamA, we performed transcriptome sequencing (RNA-Seq) analysis to determine if Bd-A treatment induces cell envelope stress. B. vulgatus ATCC 8482 was grown in liquid medium with or without a sublethal concentration of Bd-A (2 μg/ml) that still permitted growth albeit at a reduced rate. Triplicate cultures were harvested at an optical density at 600 nm (OD_600_) of 0.8 and processed for RNA-Seq analysis. A gene was considered differentially expressed (DEG) if the absolute value of the fold change of the expression level in the Bd-A-treated bacteria versus that in untreated bacteria was greater than or equal to 2 and the adjusted *P* value (*P*_adj_) for DESeq2 and the false-discovery rate (FDR) for edgeR were less than or equal to 0.05, as calculated by both statistical packages. Of the approximately 4,000 protein-encoding genes in the B. vulgatus ATCC 8482 genome, exposure to Bd-A resulted in the upregulation of 421 genes and the downregulation of 433 genes (see Table S2). Half of the 50 most downregulated genes are either *susCD* genes encoding a TonB-dependent OMP and an outer surface nutrient-binding protein involved in nutrient transport across the OM or other TonB-dependent OM receptors/transporters (Table S2; see also Fig. S6). These data suggest that the effect of intoxication with Bd-A is the downregulation of the energy-consuming processes of nutrient import. Although these TonB-dependent OM transporters were downregulated, the two TonB orthologs themselves were upregulated, one of which was the most significantly upregulated gene (Table S2; Fig. S6). Therefore, although the genes encoding numerous TonB-dependent transporters were downregulated, the organism may be redirecting TonB energetics to other OMPs, with two genes encoding porin proteins of unknown function among the top eight most highly upregulated genes. The list of upregulated genes reveals an organism that is dealing with substantial stress. Many of the genes that fall into Clusters of Orthologous Groups (COG) V “defense mechanisms” were upregulated when cells were treated with Bd-A (Table S2; Fig. S6). Four RND efflux protein systems, including all three genes of these tripartite complexes (permease, periplasmic adapter, and OM efflux protein) were upregulated. Two heat shock protein 20 (Hsp20) genes were highly upregulated as well as genes encoding the chaperones DnaK and GroEL. In addition, two periplasmic proteases that have distinct roles in maintaining OMP integrity (18) were highly upregulated. The first, BVU_0371, is a zinc metalloprotease with predicted structural similarity to BepA (PDB accession 6SAR) (19). BepA was shown to enhance the biogenesis of the essential β-barrel OMP LptD (20), which is involved in the assembly of LPS, and to also degrade misfolded LptD (20). The second is BVU_2737, which has predicted structural similarity to DegQ (PDB accession 6Z05) and also DegP. DegP and DegQ are involved in the removal of misfolded proteins in the periplasm. Therefore, two proteases involved in the removal of misfolded β-barrel OMPs are highly upregulated. Many of the DEGs in Bd-A-treated cells are similar to those identified in other organisms when BamA function is disrupted (see Discussion).

10.1128/mBio.02285-21.2 TABLE S2 Analyses of RNA-Seq data of Bd-A treated B. vulgatus ATCC 8482 versus untreated. Download Table S2, XLSX file, 2.4 MB. Copyright © 2021 Matano et al. 2021 Matano et al. https://creativecommons.org/licenses/by/4.0/ This content is distributed under the terms of the Creative Commons Attribution 4.0 International license.

10.1128/mBio.02285-21.8 FIG S6 Volcano plots of two categories of genes. (A) Of the 156 genes of B. vulgatus ATCC 8482 encoding SusCD proteins, 50 (32.05%) were downregulated and 3 (1.92%) were upregulated after 3 h of Bd-A treatment. (B) Many genes encoding proteins of the COG category V “defense mechanisms” were preferentially upregulated after Bd-A treatment. Of the 124 total genes in this category, 28 (22.58%) were upregulated, and 6 (4.84%) were downregulated. Download FIG S6, PDF file, 0.09 MB. Copyright © 2021 Matano et al. 2021 Matano et al. https://creativecommons.org/licenses/by/4.0/ This content is distributed under the terms of the Creative Commons Attribution 4.0 International license.

### *In vivo* analyses of *bamA* mutant fitness and analysis of strains from the human gut.

**FIG 5:**
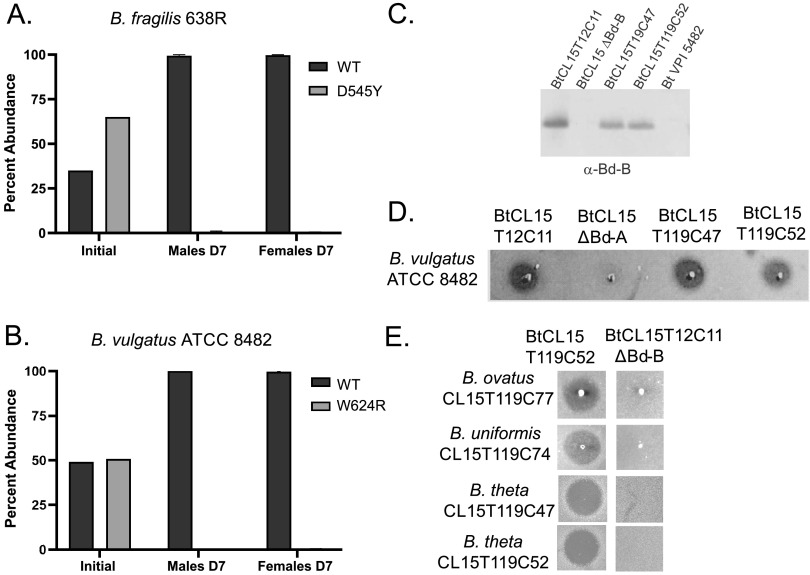
BamA mutants are fitness-attenuated *in vivo* and do not evolve in the human gut. Competitive colonization experiments in gnotobiotic mice with B. fragilis 638R and the isogenic BamA^D545Y^ mutant (A) or B. vulgatus ATCC 8482 and the isogenic BamA^W624R^ mutant (B). Mice were inoculated with a mixture of WT and respective mutant. Analysis of feces after 7 days (D7) showed the mutant strains dropped to <1% abundance, revealing a severe fitness defect. (C) Western immunoblotting results of supernatants from newly isolated B. thetaiotaomicron strains from the CL15 donor approximately 9 years after isolation of the T12 strain showed that they produced Bd-B. (D) Agar spot overlay assays show that T119 strains produce active Bd-B. (E) Isolation of several other CL15 community members at time T119, also present at T12, show continued sensitivity to Bd-B, including the producing strains. Strain designations are as follows: the first two letter indicate strain and species (Bt = B. thetaiotaomicron), The number following CL is the human subject number, which, for this study, were all isolated from individual 15, the number following the time (T) is the month of isolation after the first collection point, and the number following the C is the colony (isolate) number.

As bacteroidetocins have potential as therapeutics or in modulation of the gut microbiota, the evolution of resistance to these peptides would reduce their usefulness for such purposes. We did not detect *in vitro* growth defects in any of the *bamA* mutants, with all growing at least as well as the WT strains. However, *in vitro* growth does not indicate if the mutant strains will be fit in the mammalian gut. We tested the fitness of *bamA* mutants *in vivo* in competition with the WT strain using a Swiss-Webster gnotobiotic competitive colonization mouse model. A mixed inoculum of WT and mutant strains was introduced into germ-free mice by gavage. After 1 week, feces were collected and the proportions of each strain were quantified. This experiment was performed using B. fragilis 638R WT/BamA^D545Y^ and the B. vulgatus ATCC 8482 WT/BamA^W624R^ pairs (Fig. 5A and B). In both cases, the *bamA* mutant strains dropped to <1% abundance by 1 week, suggesting a severe fitness attenuation *in vivo*. It is therefore likely that if these mutations did arise *in vivo*, they would not reach fixation.

Next, we analyzed several isolates from the human gut to further determine if naturally evolved bacteroidetocin-resistant *bamA* mutants are present in toxin-producing strains. The *bamA* sequences of the two Bd-A-producing strains (B. vulgatus CL01T12C17 and B. vulgatus CL14T03C19) and two Bd-B-producing strains (Bacteroides ovatus CL02T12C04 and Bacteroides thetaiotaomicron CL15T12C11) do not have mutations and are sensitive to their own bacteroidetocins (7). We did not know, however, how stable bacteroidetocin-producing strains are in the human gut, if they would be lost over time, or if mutations in *bamA* would eventually arise allowing for longer-term colonization. We were able to collect a fecal sample from the original CL15 donor approximately 9 years (month 119 [T119]) after collection of an original sample (month 12 of the study [T12]), from which the original Bd-B-producing strain B. thetaiotaomicron CL15T12C11 was isolated. This T119 fecal sample was diluted and selectively plated for growth of *Bacteroidales* strains, and 63 individual colonies were screened with primers specific to the Bd-B biosynthesis region (Table S1b). Two strains were selected that produced a PCR product, and the 16S rRNA genes of these two strains were sequenced to confirm that they are B. thetaiotaomicron strains, as was the initial isolate 9 years earlier. These new strains were designated BtCL15T119C47 and BtCL15T119C52 (Bt for B. thetaiotaomicron, CL15 indicates they are from human subject 15, T119 is the month of isolation, and the number following the C is the colony or isolate number). Western immunoblot analysis showed that these later strains produced Bd-B (Fig. 5C) and that the toxin was active (Fig. 5D). Lastly, the genomes from both the original BtCL15T12C11 strain and the BtCL15T119C52 strain isolated 9 years later from the same human gut ecosystem were sequenced using SMRT (Pacific Biosciences) sequencing, which confirmed that these strains are near isogenic, with no differences in the Bd-B biosynthesis region. Importantly, even though the T119 strain still produces Bd-B, this strain did not evolve mutations in *bamA* and still self-intoxicates (Fig. 5E). From the T119 fecal sample, we also isolated several *Bacteroides* strains that were species matched with strains collected 9 years earlier (Table S1a). We used arbitrarily primed PCR (AP-PCR) to assess if these early and late species-matched stains are likely near isogenic. We identified two such pairs of strains (*B. ovatus* and Bacteroides uniformis) that produced similar PCR amplicons in all three AP-PCRs, using both the early and late strains as the template, that were different to those of nonisogenic type strains (Fig. S7). Sequence analysis of the *bamA* of these four strains revealed no differences in this gene between the early and late strains, and analyses in agar overlays showed continued sensitivity to Bd-B (Fig. 5E), despite coexisting with the Bd-B-positive B. thetaiotaomicron strain for nearly 9 years. To further evaluate if any *Bacteroidales* strains harboring Bd-A, Bd-B, or Bd-D have an alteration in the BamA sites shown in this study to confer resistance, we searched our *Bacteroidales* isolate collection of 1,434 sequenced genomes (21). We found 19 strains encoding either a Bd-A, Bd-B, or Bd-D peptide, and all BamA proteins of these strains contain the conserved aspartate, tryptophan, and asparagine residues shown in various *Bacteroidales* species to confer resistance when altered. The glycine residue of *P. bivia* that conferred resistance to Bd-D when changed to aspartate was either a glycine or the similar residue alanine in these 19 strains, and either of these two residues is normally present in the BamA of nonbacteroidetocin-harboring WT strains. These experiments suggest that organisms that evolve *bamA* mutations leading to resistance to these bacteroidetocins are not fit in the mammalian gut, further supporting the utility of the bacteroidetocins as therapeutic antibacterial molecules.

10.1128/mBio.02285-21.9 FIG S7 AP-PCR analysis of *B. ovatus* and B. uniformis T0 and T119 strains from the CL15 human gut ecosystem. Ethidium bromide (EtBr)-stained gels showing amplicons of AP-PCR using one of three primers (I, II, and III) for *B. ovatus* strains CL15T00C12 and CL15T119C77 (A) and B. uniformis strains CL15T00C17 and CL15T119C74 (B). The type strain of each species is used as a control to show how these AP-PCRs differentiate strains of the same species. Download FIG S7, PDF file, 0.1 MB. Copyright © 2021 Matano et al. 2021 Matano et al. https://creativecommons.org/licenses/by/4.0/ This content is distributed under the terms of the Creative Commons Attribution 4.0 International license.

## DISCUSSION

BamA is a β-barrel OMP containing periplasmic polypeptide translocation-associated (POTRA) domains responsible for binding chaperoned proteins in the periplasm and a β-barrel domain consisting of 16 β-strands that are involved in the final folding and inserting of OMPs into the OM (reviewed reference 12). The BamA proteins of Escherichia coli and Neisseria gonorrhoeae each contain seven extracellular loops (eLs), of which loop 6 (eL6) is highly conserved and contains the (V/I)RG(F/Y) motif which interacts with other residues in the β-strands of the barrel wall (14). The surface-exposed eL6 of E. coli BamA is necessary for its activity as it undergoes a conformational change during the OMP assembly reaction (22). The interaction of residues of eL6 with the interior of the nascent β-barrel wall is an important step in the folding of OMPs (23). Recent studies have shown that binding of diverse molecules to the eLs of BamA inhibits its function, leading to cell death. One example is the lectin-like bacteriocins (LlpAs) produced by Pseudomonas that initially interact with the common polysaccharide antigen of the LPS of similar species (24). This interaction is predicted to stabilize or facilitate its interaction with eL6 of BamA, inhibiting its function (25). Another example is the ribosomally synthesized modified small peptide darobactin produced by Photorhabdus khanii, which inhibits BamA activity, resulting in cell death (15). In contrast to Bd-A and LlpA which bind the surface of BamA, darobactin mimics the recognition signal of native substrates and binds the lateral gate of BamA of E. coli (26) near the periplasmic interface. Another study showed that the small molecule compound MRL-494 functions at the outer surface of E. coli by binding to BamA and inhibiting OMP biogenesis (16). In addition, a monoclonal antibody that binds eL4 of the BamA of E. coli inhibits OMP folding activity and is bactericidal (27).

The present study strongly suggests that Bd-A not only binds BamA but also inhibits its function. The only mutations identified that render *Bacteroidales* strains resistant to Bd-A killing were in *bamA*. We also showed that fluorescently labeled Bd-A binds to the surfaces of WT cells but not to those of isogenic strains with *bamA* mutations. In addition, several Bd-A-mediated effects in treated cells were similar to those described in other Gram-negative bacteria treated with molecules that inhibit BamA function. For example, the kinetics of killing by Bd-A is similar to that by darobactin, which was shown to lead to a two-log decrease in CFU by 2 h (15). Darobactin-mediated cellular morphological changes included membrane blebbing, cell rounding, and subsequent bursting of cell membranes, as we show here in Bd-A-treated cells. Another study using CRISPRi knockdown of *bamA* of Pseudomonas aeruginosa also showed cellular rounding and eventual cell lysis (17). Lastly, the transcriptomics data of Bd-A-treated cells demonstrate a response indicative of outer membrane stress. In *Proteobacteria*, inhibition of BamA function led to transcriptional responses where the σ^E^ stress response regulon was upregulated, including upregulation of *degP* (15) encoding the periplasmic protease involved in degrading misfolded proteins. Similarly, quantitative proteomic analysis revealed increased levels of DegP in E. coli treated with MRL-494 (16). Here, treatment of B. vulgatus with Bd-A resulted in the increase of transcripts encoding the two predicted periplasmic proteases, DegQ/P and BepA, involved in removing misfolded OMPs.

The BamA proteins of E. coli and B. vulgatus are not highly similar in their primary sequences, especially in eL3. The eL3 of B. vulgatus is 44 residues long, compared to that E. coli, which is 12 residues. Of these 44 residues of the B. vulgatus BamA, 22 are tyrosines and asparagines, residues rarely seen in low-complexity regions of prokaryotic proteins (28). Four of the seven *bamA* mutations that lead to Bd-A resistance in different genera of *Bacteroidales* altered the same aspartate residue predicted to be at the interface of the transmembrane domain and eL3. The other three *bamA* mutations that conferred Bd-A resistance all altered residues at the predicted transmembrane interface with eL2 or eL4. Mutants resistant to the LlpA bacteriocin also mapped to three different eLs of BamA (24), suggesting a three-dimensional interaction motif. Of the 38 *Bacteroidales* strains that have been tested with Bd-A, no strains that lack the aspartate residue of eL3 are susceptible to Bd-A, and all but one strain that contains the aspartate residue at this site are sensitive to Bd-A. It is our prediction, based on the fact that different bacteroidetocins target different strains and species, that different residues at the transmembrane interfaces of these eLs will dictate specificity. Once the molecular and structural interactions between the bacteroidetocins and BamA are elucidated, it should be possible to rationally design peptides to target the BamA of specific *Bacteroidales* pathogens, such as Porphyromonas gingivalis, for which we have not yet identified a toxic bacteroidetocin.

Competitive colonization experiments demonstrate that Bd-A escape mutants selected in our *in vitro* assay are not fit to compete in the mammalian gut. Even more relevant are the findings that Bd-producing *Bacteroidales* strains in the human gut do not contain *bamA* mutations and, in the one ecosystem we analyzed, were stable members of the human gut microbiota. We previously showed that Bd-B peptide is present in the stool of mice monocolonized with BtCL15T12C11 (7), showing that this strain produces Bd-B in the mammalian gut. These data raise the question of how a strain that has the ability to self-intoxicate would be fit for gut colonization. It is possible that the organism may upregulate the expression of protective genes *in vivo* that are not expressed *in vitro.* It is also possible that the toxin is only produced by a small proportion of cells. In addition, the bacteroidetocins may be produced only at high cell density, which may explain how the BtCL15 strains can grow as a lawn when inoculated at low density, such as in top agar of overlay assays. Future analysis of the regulation of toxin production and subsequent secretion from the cell will likely reveal important ecological properties of bacteroidetocin production and whether the bacteroidetocins confer an as-yet-unrecognized benefit to the producing cell.

Unlike previously discovered natural and synthetic inhibitors of BamA, the bacteroidetocins uniquely target species of *Bacteroidetes*. The data support that the bacteroidetocins are bactericidal and that resistant mutants are not fit *in vivo*, highlighting their therapeutic potential against pathogenic *Bacteroidales* species, in particular, pathogenic *Prevotella* species. Here, we show that Bd-A targets all four species of pathogenic vaginal *Prevotella* implicated in bacterial vaginosis, *P. bivia*, P. amnii, P. timonensis, and P. disiens. The high degree of treatment failure and subsequent recurrence of B. vulgatus necessitate new treatment strategies. The data support that bacteroidetocins could be important as a next-generation targeted treatment.

## MATERIALS AND METHODS

### Bacterial strains and growth.

All strains are listed in Table S1a in the supplemental material. *Bacteroides* and *Parabacteroides* spp. were grown on supplemented brain heart infusion (BHIS) plates or were cultured in basal liquid medium (7). All other strains were grown on Brucella blood agar plates containing hemin and vitamin K (Remel R01255) and in liquid tryptic soy broth (TSB) containing per liter 30 g TSB, 5 g yeast extract, and 0.5 g cysteine-HCl, with the addition of hemin and vitamin K_1_ following autoclaving. When appropriate, antibiotics were added at the following concentrations: erythromycin, 10 μg/ml; tetracycline, 6 μg/ml. E. coli cells were grown in LB broth or on plates with 100 μg/ml carbenicillin where appropriate.

### Agar spot overlay assays.

*Parabacteroides* and most *Bacteroides* strains were grown on BHIS agar plates, and *Prevotella*, *Porphyromonas* and *Dysgonomonas* as well as *Bacteroides abscessus*, *B. coprophilus*, and *B. plebius* were grown on Brucella blood plates. Synthesized mature Bd peptides were purchased from LifeTein (Somerset, NJ). Strains to test for sensitivity in overlays were inoculated into 5 ml of prereduced basal or tryptic soy broth (TSB) and grown until they reached mid-log phase; 50 μl of bacteria was added to 5 ml of 0.75% top agar (either BHIS or TSB based) and poured over plates. After the top agar had solidified, 10 μl of a 100-ng/μl solution of synthesized mature Bd-A, Bd-A-FAM, or Bd-D dissolved in deionized (DI) water or 10 μl of DI water (for controls) was spotted onto the top and allowed to dry. Testing for sensitivity to Bd-A and Bd-B produced from *Bacteroides* strains was performed as previously described (7). Zones of growth inhibition were analyzed after 24 to 72 h, depending on the rate of growth of the strain.

### Effects of Bd-A on growth in broth.

Bacteria were grown anaerobically to mid-log phase in either basal or TSB and then diluted to an OD_600_ of 0.1; 10 μl of this culture was added to 100 μl of fresh medium containing 1 μg of bacteroidetocin A in at least triplicate wells of a 96-well plate. Control wells were inoculated with sterile DI water, and OD_600_ was recorded every 10 min for 18 to 48 h using an Eon high-performance microplate spectrophotometer (BioTek Instruments, Winooski, VT). Data were recorded every 10 min and plotted as the average of at least three biological replicates ± the standard deviation (SD) as calculated by Prism version 8.0.1 for 64-bit Windows (GraphPad Software, San Diego, CA).

### Creation of BamA site mutants.

Site mutations were created in *bamA* using the pLGB13 counterselection vector (29). Primers were designed to amplify B. fragilis 638R BamA^D545Y^, B. vulgatus ATCC 8482 BamA^D546Δ^, and B. vulgatus ATCC 8482 BamA^W624R^ (Table S1b). PstI- and BamHI-digested pLGB13 was joined with the mutant *bamA* products using NEBuilder (New England BioLabs). DNA was transformed into E. coli S17 λpir, and colonies arising on carbenicillin plates were tested by PCR; the plasmids were then sequenced. The plasmids were transferred by conjugation from E. coli to the WT *Bacteroides* strain, and cointegrates were selected on erythromycin plates. Cointegrates were passaged in nonselective medium and then plated on BHIS plates containing 100 ng/ml anhydrotetracycline, selecting for double-crossover recombinants. Mutants were confirmed by sequencing *bamA*.

### Competitive colonization assay in gnotobiotic mice.

Mouse experiments were approved by the Institutional Animal Care and Use Committee (IACUC), Brigham and Women’s Hospital. WT and mutant isogenic strains made tetracycline resistant (Tet^r^) or erythromycin resistant (Em^r^), respectively, were mixed in a 1:1 ratio, and 200 μl was gavaged into each of three male and three female Swiss-Webster germ-free mice housed separately under gnotobiotic conditions in Opti-mouse cages. Seven days after inoculation, fecal samples were collected, diluted, an plated on nonselective BHIS plates, and, after 2 days, samples were replica plated to tetracycline and erythromycin plates. WT and mutant colonies were quantified after 2 days.

### Western immunoblot analysis.

Bacteria from overnight cultures were boiled in lithium dodecyl sulfate (LDS) sample buffer, and the equivalent of 5 μl of bacterial lysate was separated by electrophoresis using NuPAGE 12% 2-(*N*-morpholino)ethanesulfonic acid (MES) polyacrylamide gels (Invitrogen). The contents of the gels were transferred to polyvinylidene difluoride membranes, blocked with skim milk, and probed with anti-Bd-B antibodies as previously described (7).

### Additional methods.

Text S1 contains the methods for widefield fluorescence and transmission electron microscopy, RNA-Seq analyses, isolation and sequencing of BtCL15 strains, and arbitrarily primed PCR analyses.

10.1128/mBio.02285-21.10 TEXT S1 Methods for wide-field fluorescence microscopy, transmission electron microscopy, RNA-Seq analyses, isolation and sequencing of BtCL15 strains, and arbitrarily primed PCR analyses. Download Text S1, DOCX file, 0.04 MB. Copyright © 2021 Matano et al. 2021 Matano et al. https://creativecommons.org/licenses/by/4.0/ This content is distributed under the terms of the Creative Commons Attribution 4.0 International license.

### Data availability.

RNA-seq data are provided in Table S2. The BioProject accession number for the genome sequences of BtCL15T12C11 and BtCL15T119C52 is PRJNA728094.
