# Supplementary material for: Bacteroidetocins Target the Essential Outer Membrane Protein BamA of Bacteroidales Symbionts and Pathogens
Source: mBio. 2021 Sep 14;12(5):e02285-21. doi: 10.1128/mBio.02285-21 (PMC8546649; doi:10.1128/mBio.02285-21)
Supplement: FIG S1 [file mbio.02285-21-sf001.pdf]

Supplemental Figures

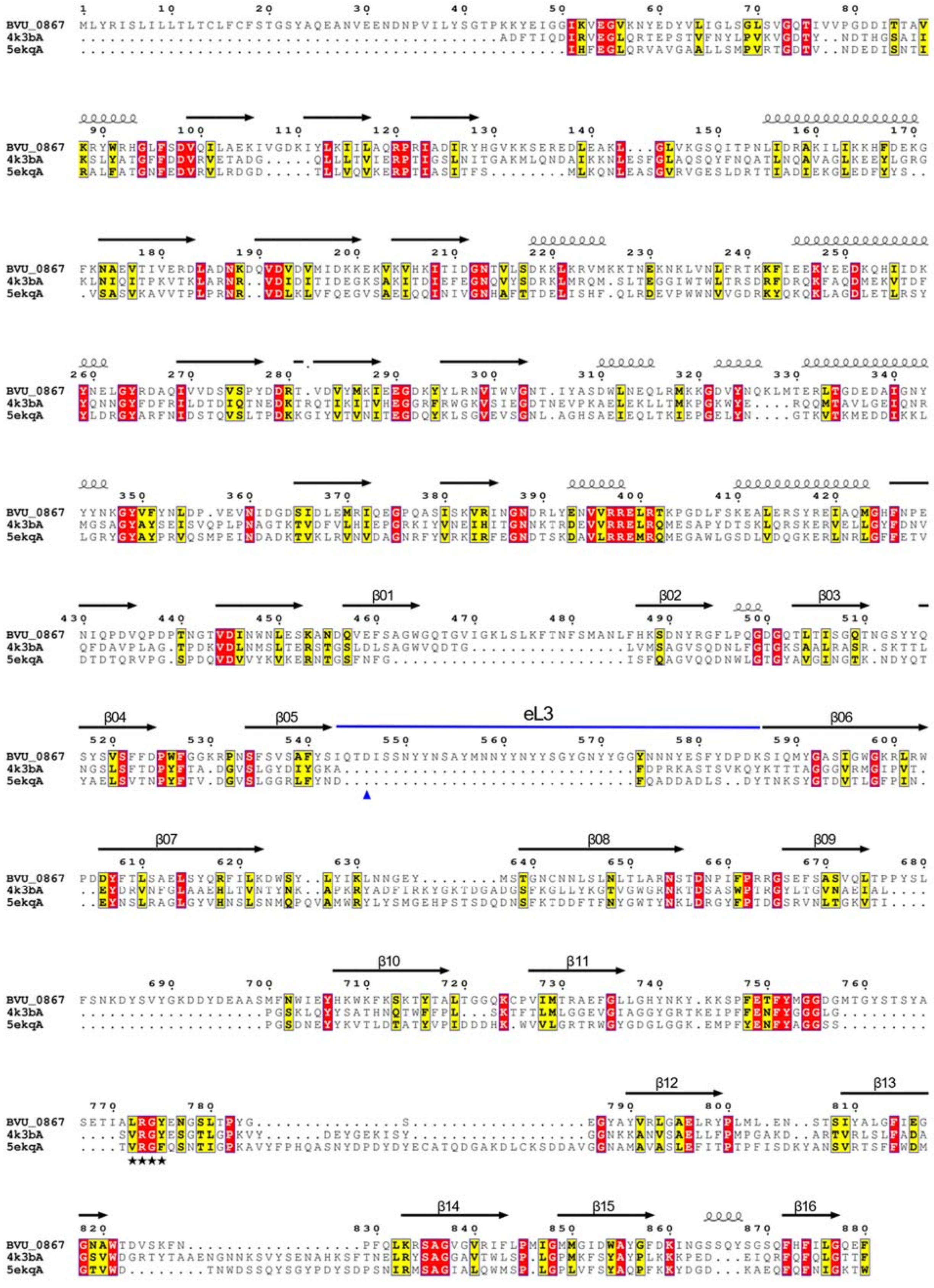

Figure S1a. Alignment of *B. vulgatus* 8482 BamA with BamA sequences of *N. gonorrhoeae* F0103 and *E. coli* K12. Phyre2 alignment of BamA (BVU_0867) to PDB sequences of *N. gonorrhoeae* (43kbA) and *E. coli* K12 (5ekqA). Predicted secondary structure from 3D model of BVU_0867 is displayed above the sequence. Percent identity is 25% for BamA of *N. gonorrhoeae* and 24% for *E. coli*. Conserved residues are highlighted in red, similar residues are highlighted in yellow. The 16 predicted β-strands of the β-barrel are numbered, and eL3 is noted with a blue bar. Loop eL3 is longer in *B. vulgatus* (Bv) BamA than in the other two species. The site of eL3D is noted by a blue arrowhead below the sequences, and the four starred residues indicate the conserved (V/I)RG(F/Y) motif. Illustration was generated using ESPript 3.0.[1]. Horizontal arrows indicate β-strands and horizontal helical symbols represent α-helices.

1. Robert, X. and P. Gouet, *Deciphering key features in protein structures with the new ENDscript server.* Nucleic Acids Research, 2014. **42**(W1): p. W320-W324.

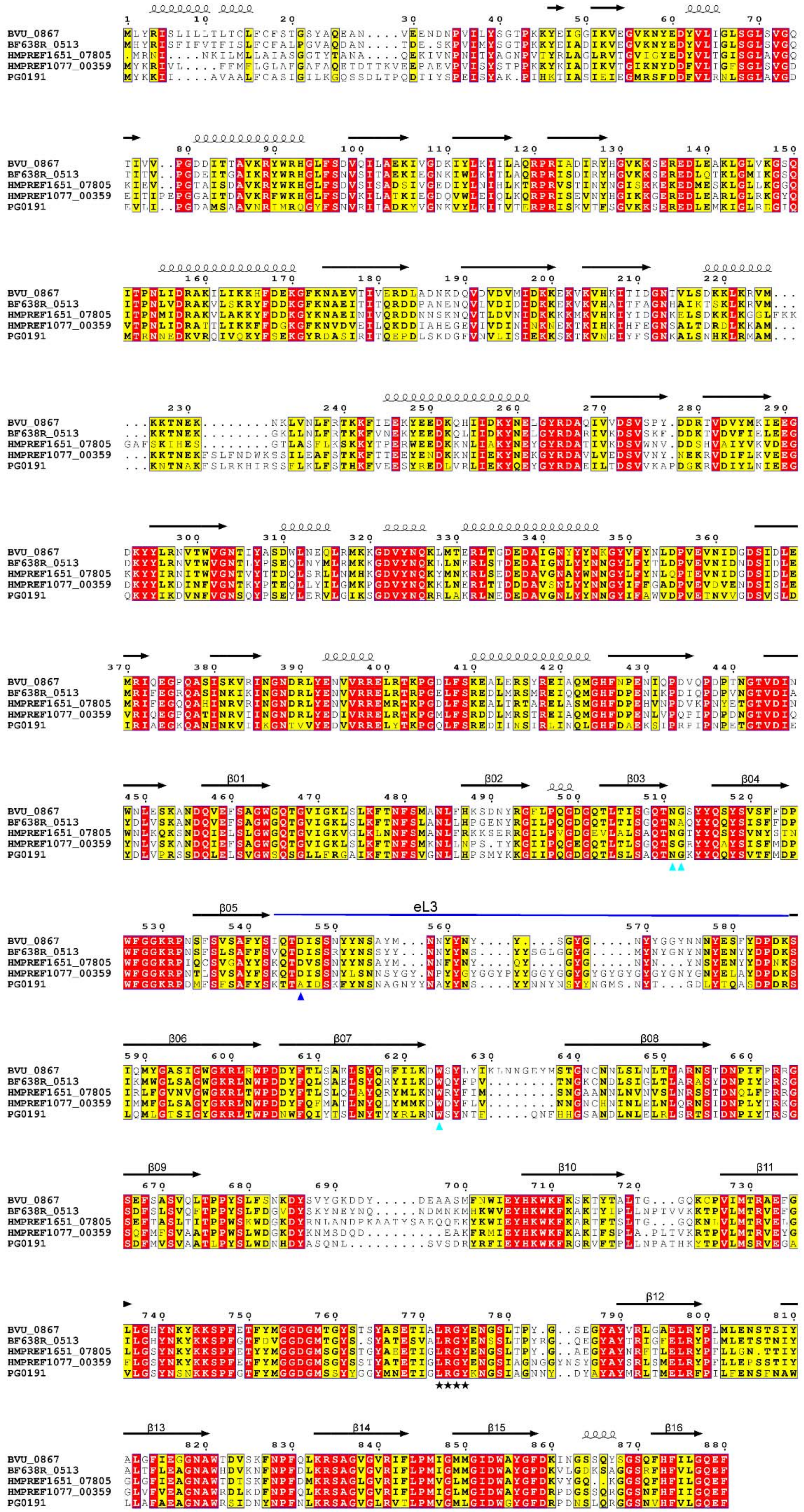
BVU_0867
BF638R_0513
HMPREF1651_07805
HMPREF1077_00359
PG0191
β01
β02
β03
β04
β05
eL3
β06
β07
β08
β09
β10
β11
β12
β13
β14
β15
β16

Figure S1b. Alignment of *B. vulgatus* 8482 BamA with BamA sequences of four other Bacteroidales species. Alignment of Bv BamA (BVU_0867) to *B. fragilis* 638R (BF638R_0513), *Pr. bivia* DNF00188 (HMPREF1651_07805), *P. johnsonii* CL02T12C29 (HMPREF1077_00359) and *Po. gingivalis* W83 (PG0191). The predicted secondary structure from the 3D model of BVU_0867 is displayed above the sequence. Conserved residues are highlighted in red, similar residues are highlighted in yellow and the four starred residues indicate the conserved (V/I)RG(F/Y) motif. The 16 predicted β-strands of the β-barrel are numbered and eL3 is noted with a blue bar. The site of eL3D is noted by a blue arrowhead below the sequences; other mutation sites are noted with cyan arrowhead. Illustration was generated using ESPript 3.0.
