# Supplementary material for: Bacteroidetocins Target the Essential Outer Membrane Protein BamA of Bacteroidales Symbionts and Pathogens
Source: mBio. 2021 Sep 14;12(5):e02285-21. doi: 10.1128/mBio.02285-21 (PMC8546649; doi:10.1128/mBio.02285-21)
Supplement: FIG S2 [file mbio.02285-21-sf002.pdf]

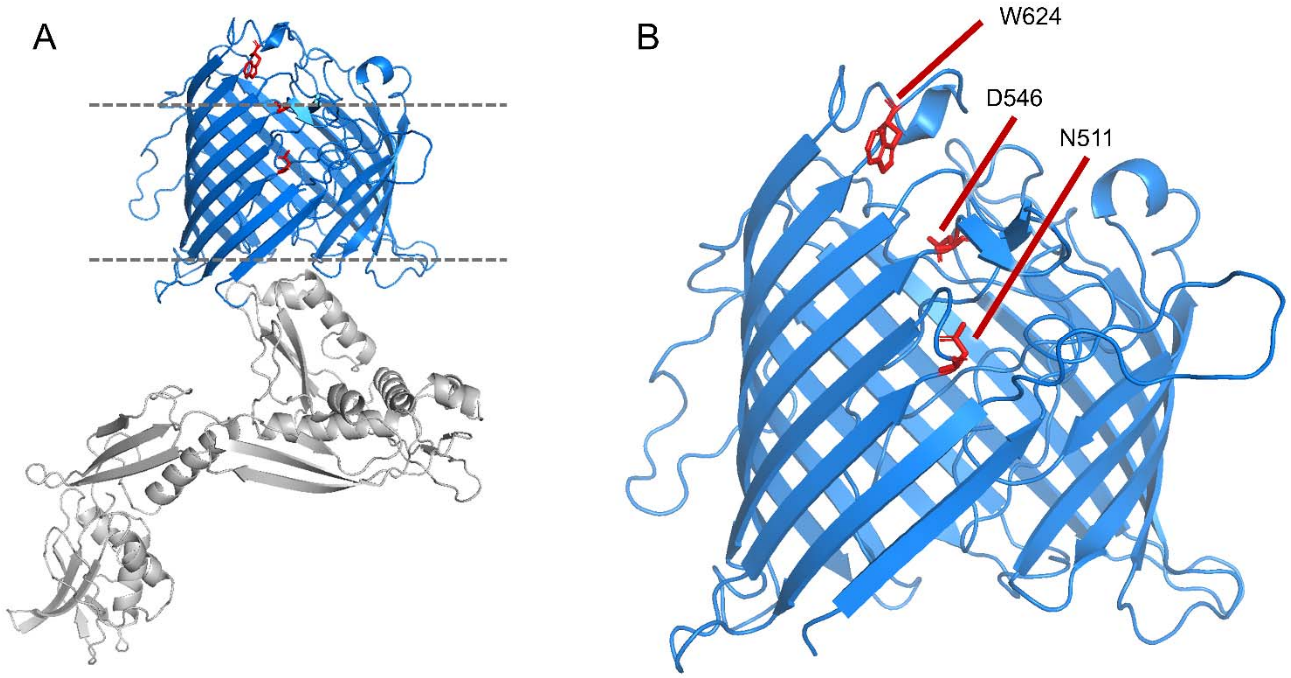

Figure S2. Mapping Bd-A resistant mutant sites onto a predicted BamA structure of *B. vulgatus* ATCC 8482*.* A. BamA structure predicted using Phyre2 and mapping to the crystal structure of *Neisseria gonorrhoeae* FA 1090 (4K3B). Periplasmic region including the POTRA domains is shown in grey, β-barrel domain is shown in blue. **B.** Close up of β-barrel domain. The sites of two Bv mutations (W624R and D546Δ) as well as the equivalent location of the *P. johnsonii* N520S mutation in the C-terminal portion are shown in red.

Kelley, L.A., et al., *The Phyre2 web portal for protein modeling, prediction and analysis.* Nature Protocols, 2015. **10**(6): p. 845-858.
