# Supplementary material for: Bacteroidetocins Target the Essential Outer Membrane Protein BamA of Bacteroidales Symbionts and Pathogens
Source: mBio. 2021 Sep 14;12(5):e02285-21. doi: 10.1128/mBio.02285-21 (PMC8546649; doi:10.1128/mBio.02285-21)
Supplement: FIG S3 [file mbio.02285-21-sf003.pdf]

```
Pr. oris DSM 18711     MNNIKKAFFILAAGMTCSVPAFAQDKILHPDISYAGTPRSCIIGGINISGIEGYEDYMLT 60
Pr. oris F0302         MNNIKKAFFILAAGMTCSVPAFAQDKILHPDISYAGTPRSCIIGGINISGIEGYEDYMLT 60
                       ************************************************************

Pr. oris DSM 18711     GISGLQVGGQEITVPGTEITNAVKRYWRHGLFSDVQISADSIVGRKIYLHIALKTRPRVSV 120
Pr. oris F0302         GISGLQVGGQEITVPGTEITNAVKRYWRHGLFSDVQISADSIVGRKIYLHIALKTRPRVSV 120
                       ************************************************************

Pr. oris DSM 18711     INYEGLKKSEREDMEKKLGLLKGSQITPNMIDRAKILAKKYFDDKGFKNAEITINQRDDV 180
Pr. oris F0302         INYEGLKKSEREDMEKKLGLLKGSQITPNMIDRAKILAKKYFDDKGFKNAEITINQRDDV 180
                       ************************************************************

Pr. oris DSM 18711     ASKGNVILDVIVDKKEKMKVRSIIIEGNKALTSSKIKGGLFKKGAFAKIHEAGKLSSFLK 240
Pr. oris F0302         ASKGNVILDVIVDKKEKMKVRSIIIEGNKALTSSKIKGGLFKKGAFAKIHEAGKLSSFLK 240
                       ************************************************************

Pr. oris DSM 18711     AKKFTPERWATDKKNLIEKYNEYGYRDATLLKDSVWNVDPKHVDIYVKIDEGQKYYLRNI 300
Pr. oris F0302         AKKFTPERWATDKKNLIEKYNEYGYRDATLLKDSVWNVDPKHVDIYVKIDEGQKYYLRNI 300
                       ************************************************************

Pr. oris DSM 18711     TWVGNTVYSTLQLNSILGMKKGDVYNQKLLDKRVSKDEDAAANLYYNHGYVFSSINPTEV 360
Pr. oris F0302         TWVGNTVYSTLQLNSILGMKKGDVYNQKLLDKRVSKDEDAAANLYYNHGYVFSSINPTEV 360
                       ************************************************************

Pr. oris DSM 18711     NIDGDSIDLEMRVTEGPQAYLSHVRINGNTRLYENVIRRELRTKPGDLFSREALMRSLRE 420
Pr. oris F0302         NIDGDSIDLEMRVTEGPQAYLSHVRINGNTRLYENVIRRELRTKPGDLFSREALMRSLRE 420
                       ************************************************************

Pr. oris DSM 18711     IQSMGHFDPEKSSQPDVKPNPEDGTVDINYNLEQKSNDQIEFSLGWGQTGVIGKIGLKLN 480
Pr. oris F0302         IQSMGHFDPEKSSQPDVKPNPEDGTVDINYNLEQKSNDQIEFSLGWGQTGVIGKIGLKLN 480
                       ************************************************************

Pr. oris DSM 18711     NFSMANLFRKNKEHRGIMPIGDGEVLSLSAQTNGSYYQSYNASYSTNWLGGKRPNQFSVG 540
Pr. oris F0302         NFSMANLFRKNKEHRGIMPIGDGEVLSLSAQTNGSYYQSYNASYSTNWFGGKRPNQFSVG 540
                       ************************************************:***********

Pr. oris DSM 18711     LYFSKMTDVSSSYYNDAYRRNYLNSYYGGYGYGYGNYNNNYYENYYDPDTYMRMYGISLG 600
Pr. oris F0302         LYFSKLTGLSNSYYNDAYRQNYLNSYYGGYGYGYGNYNNNYYENYYDPDTYMRMYGISLG 600
                       *****:*.:*.********:****************************************

Pr. oris DSM 18711     WGKRLRWPDDYFVLSAQLAYQRYQLKNWKYFLMNNGAANNLNLSISLSRTSTDNPLFPRR 660
Pr. oris F0302         WGKRLRWPDDYFVLSAQLAYQRYQLKNWKYFLMNNGAANNLNLSISLSRTSTDNPLFPRH 660
                       ***********************************************************:

Pr. oris DSM 18711     GSEFTASVTLTPPWSVWSHKDYKNLAMNPYSPTYSAEQQEKYRWVEYHKWKFKAKTYTAL 720
Pr. oris F0302         GSEFTASVTLTPPWSVWSHKDYKNLAMNPYSPTYSAEQQEKYRWVEYHKWKFKAKTYTAL 720
                       ************************************************************
```

```
Pr. oris DSM 18711     SGGQKCFVLMTRIEMGLLGSYNKYKKSPFETYYMGGDGMSGTSSSYADETIGLRGYENGS 780

Pr. oris F0302         SGGQKCFVLMTRIEMGLLGSYNKYKKSPFETYYMGGDGMSGTSSSYADETIGLRGYENGS 780
                       ************************************************************

Pr. oris DSM 18711     LAYNGYAYDRFTLELRYPFLLGNTTIYGLGFVEAGNAWSDTKYFNPFDMKRSAGLGVRIY 840

Pr. oris F0302         LAYNGYAYDRFTLELRYPFLLGNTTIYGLGFVEAGNAWSDTKYFNPFDMKRSAGLGVRIY 840
                       ************************************************************

Pr. oris DSM 18711     LPMVGLMGIDWAYGFDKDNVNKKKGGSQFHFILGQEF      877

Pr. oris F0302         LPMVGLMGIDWAYGFDKDNVNKKKGGSQFHFILGQEF      877
                       *************************************
```

Figure S3. Comparison of the BamA sequences of two *Prevotella oris* strains that differ in Bd-A sensitivity. Alignment of BamA sequences of *Pr. oris* DSM 18711 and *Pr. oris* F0302. The sequences are nearly identical except for the seven amino acids highlighted in yellow. Critically, DSM 18711 has eL3D (shown in red at position 548) but F0302 has a G at this site, and three other resides in this region are different. The two residues (N513 and W628) shown in other strains to confer resistance to Bd-A are highlighted in turquoise and are identical between the BamA of the two strains.
