# Supplementary material for: Bacteroidetocins Target the Essential Outer Membrane Protein BamA of Bacteroidales Symbionts and Pathogens
Source: mBio. 2021 Sep 14;12(5):e02285-21. doi: 10.1128/mBio.02285-21 (PMC8546649; doi:10.1128/mBio.02285-21)
Supplement: FIG S4 [file mbio.02285-21-sf004.pdf]

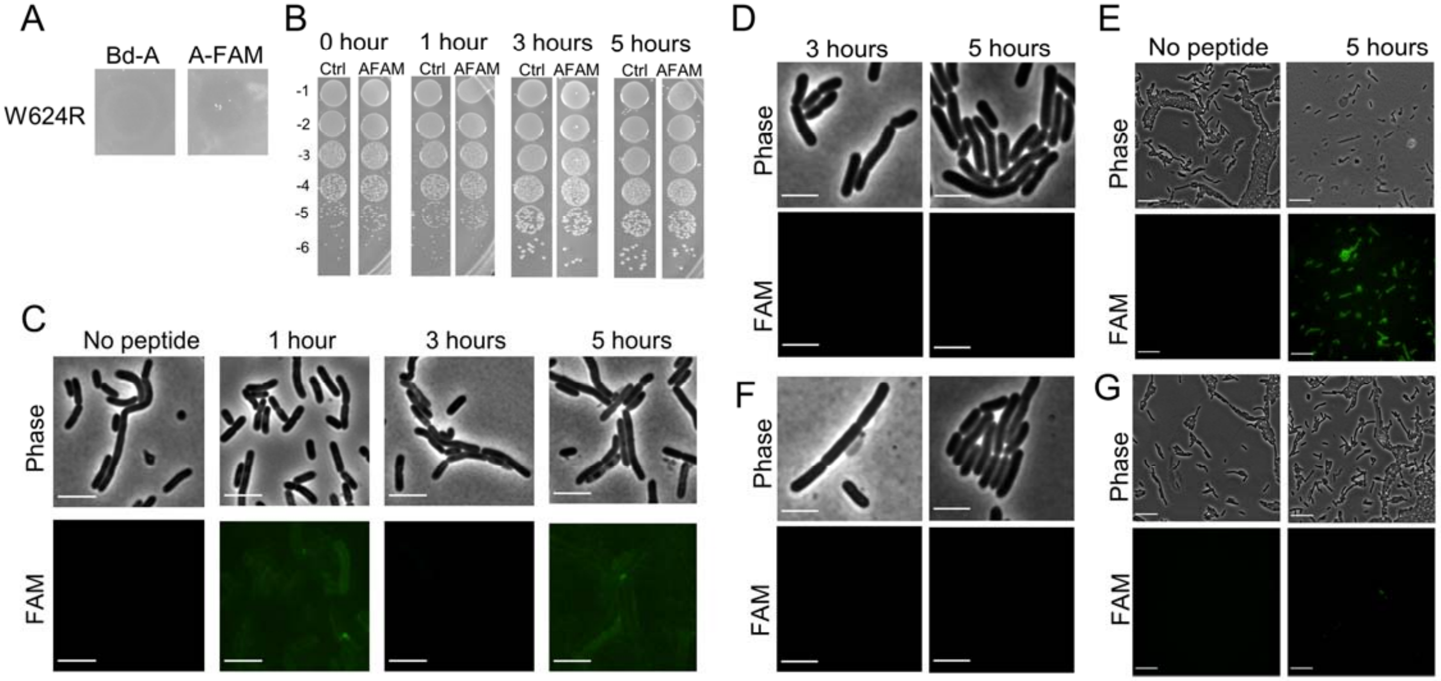

Figure S4. **Bd-A-FAM treatment of *B. vulgatus* ATCC 8482 BamA^{W624R} and Bd-A-FAM control images.** **A.** Overlays of *B. vulgatus* ATCC 8482 BamA^{W624R} show that Bd-A-FAM does not inhibit growth. **B.** *B. vulgatus* ATCC 8482 BamA^{W624R} treated with 10 ng/µl of BD-A-FAM continues to grow over time similarly to the untreated control. **C.** Fluorescence microscopy of the *B. vulgatus* ATCC 8482 BamA^{W624R} mutant with A-FAM. Images show that cells maintain normal shape upon treatment and little association of Bd-A-FAM with cells. Bars are 5 µm. **D.** Untreated *B. vulgatus* ATCC 8482 wild-type (WT) at three and five hours. These photos are controls for the three and five hour time points shown in Fig. 4 in the main text. Bars are 5 µm. **E.** An uncropped image of *B. vulgatus* WT untreated and treated with 10 ng/µL of Bd-A for 5 hours. The treated sample shows many rounded cells brightly fluorescing. Bars are 20 µm. **F.** Untreated *B. vulgatus* ATCC 8482 BamA$^{D546\Delta}$ mutant cells at three and five hours. These photos are controls for the three and five hour time points shown in Fig. 4 in the main text. Bars are 5 µm. **G.** Uncropped image of *B. vulgatus* BamA$^{D546\Delta}$ mutant untreated and treated with 10 ng/µL of Bd-A-FAM. Images show that the mutant strain does not have visibly bound Bd-A-FAM, unlike the treated wild-type sample. Bars are 20 µm.
