# Supplementary material for: Bacteroidetocins Target the Essential Outer Membrane Protein BamA of Bacteroidales Symbionts and Pathogens
Source: mBio. 2021 Sep 14;12(5):e02285-21. doi: 10.1128/mBio.02285-21 (PMC8546649; doi:10.1128/mBio.02285-21)
Supplement: FIG S5 [file mbio.02285-21-sf005.pdf]

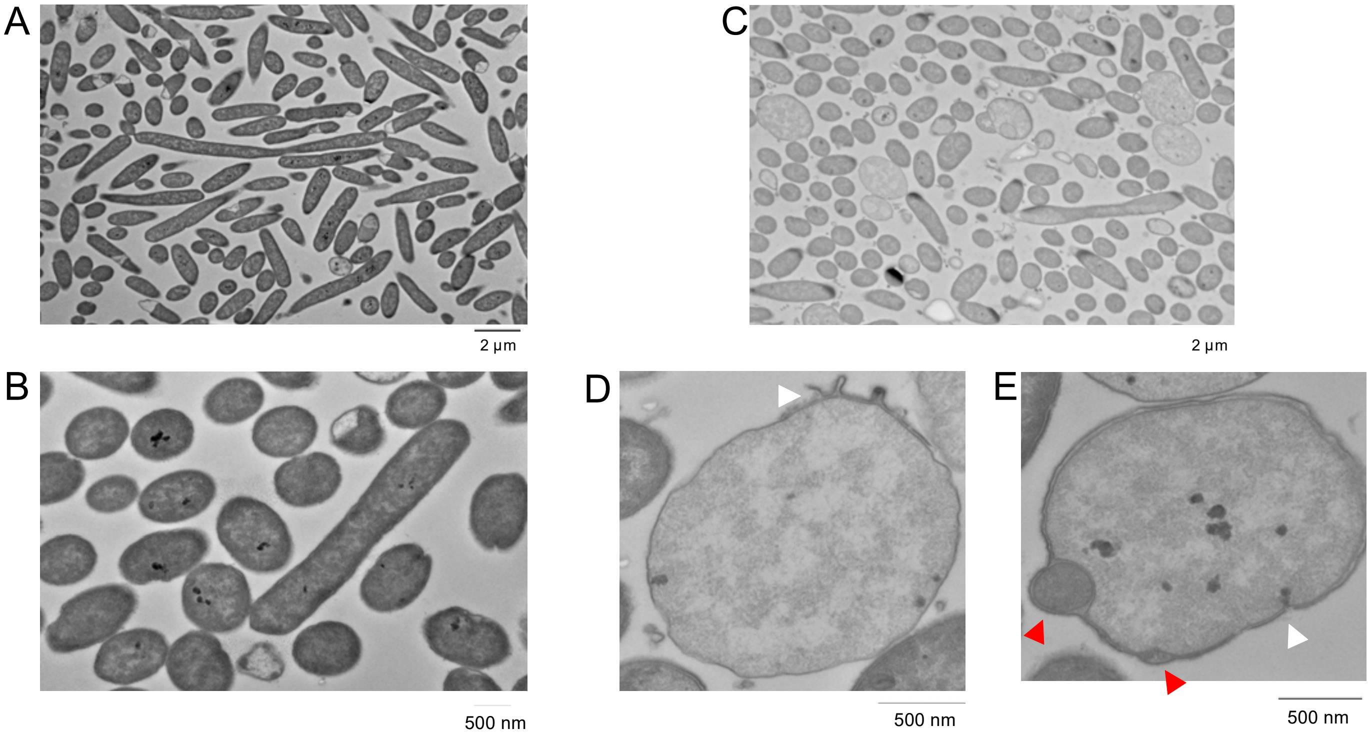

Figure S5: TEM imaging of *B. vulgatus* ATCC 8482 treated with Bd-A shows rounding and outer membrane defects. **A.** Cross section of fixed *B. vulgatus* ATCC 8482 WT cells. **B.** Close up of healthy WT cells. **C.** Cross section of fixed *B. vulgatus* ATCC 8482 treated with Bd-A. **D-E.** Higher magnification of large abnormal cells. Cells show breaks and loss of outer cell membrane (white arrows). Cells also show abnormal accumulation of debris between inner and outer membranes (red arrows).
