# Supplementary material for: Bacteroidetocins Target the Essential Outer Membrane Protein BamA of Bacteroidales Symbionts and Pathogens
Source: mBio. 2021 Sep 14;12(5):e02285-21. doi: 10.1128/mBio.02285-21 (PMC8546649; doi:10.1128/mBio.02285-21)
Supplement: FIG S6 [file mbio.02285-21-sf006.pdf]

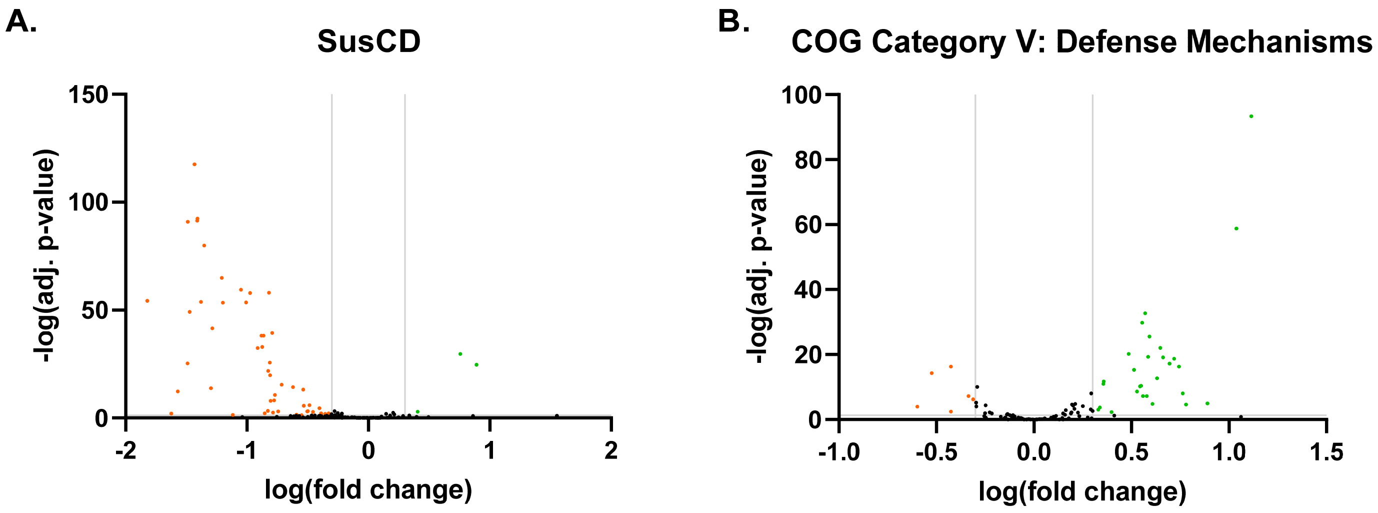

Figure S6. Volcano plots of two categories of genes. A. Of the 156 genes of *B. vulgatus* ATCC 8482 encoding SusCD proteins, 50 (32.05%) are downregulated and 3 (1.92%) are upregulated after 3 hours of Bd-A treatment. **B.** Many genes encoding proteins of the COG category V "defense mechanisms" are preferentially upregulated after Bd-A treatment. Of the 124 total genes in this category, 28 (22.58%) are upregulated, and 6 (4.84%) are downregulated.
