# Supplementary material for: Bacteroidetocins Target the Essential Outer Membrane Protein BamA of Bacteroidales Symbionts and Pathogens
Source: mBio. 2021 Sep 14;12(5):e02285-21. doi: 10.1128/mBio.02285-21 (PMC8546649; doi:10.1128/mBio.02285-21)
Supplement: FIG S7 [file mbio.02285-21-sf007.pdf]

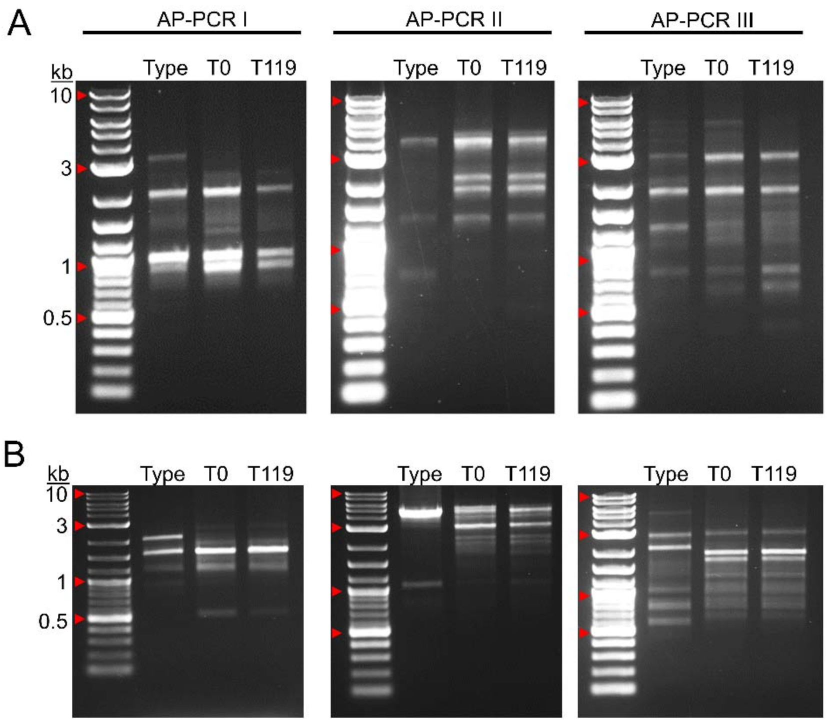

Figure S7. AP-PCR analysis of *B. ovatus* and *B. uniformis* T0 and T119 strains from the CL15 human gut ecosystem. EtBr-stained gels showing amplicons of AP-PCR using one of three primers (I, II and III) for **A.** *B. ovatus* strains CL15T00C12 and CL15T119C77 and **B.** *B. uniformis* strains CL15T00C17 and CL15T119C74. The type strain of each species is used as a control to show how these AP-PCRs differentiate strains of the same species.
