# Supplementary material for: Bacteroidetocins Target the Essential Outer Membrane Protein BamA of Bacteroidales Symbionts and Pathogens
Source: mBio. 2021 Sep 14;12(5):e02285-21. doi: 10.1128/mBio.02285-21 (PMC8546649; doi:10.1128/mBio.02285-21)
Supplement: TEXT S1 [file mbio.02285-21-s0001.docx]

**Supplemental Methods**

BamA alignments and topology predictions

Clustal Omega multiple sequence alignment software was used to align BamA sequences using standard parameters. 3D structure prediction was performed using Phyre2 (1) and modeled using the crystal structure of the BamA of *Neisseria gonorrhea* FA 1090 (PDB accession 4K3B) (2).

Widefield fluorescence microscopy

Bd-A labeled with FAM (Bd-A-FAM) at the C-terminus was purchased from LifeTein (Somerset, NJ) as a lyophilized powder. Bd-A-FAM was suspended in DI water, filter sterilized using a 0.2 µm PES filter, and the protein concentration was measured with a Qubit Protein Assay Kit (Invitrogen). FM4-64 was purchased from Invitrogen (T-3166) and was dissolved in water to 1 mg/mL. WT and mutant strains were inoculated into fresh pre-reduced basal and grown anaerobically for several hours until an OD_600_ of 0.2. At that time, 10 µL containing 1 µg of peptide A-FAM or 10 µL of DI water as a control was added to 100 µL of WT and mutant cultures. At various time points, 10 µL of treated or control cultures were spotted on phosphate buffered saline (PBS) agarose (1.2%) pads on microscope slides. For comparison with FM6-64, agarose pads contained 1 µg/mL FM4-64. After addition of the culture aliquot to agar pads, the samples were allowed to dry, a cover slip was placed on top, and silicone was piped around the edges to seal the sample from desiccation and entry of oxygen. Samples were imaged using a Nikon Ti Inverted Microscope with Motorized Stage and Plan Apo 100x/1.40 Oil Ph3 DM objective. A Lumencore SpectraX LED light source was used, and GFP (49002) and mCherry (49008) Chroma ET filter cubes in a motorized filter turret were utilized. The GFP configuration was used to image Bd-A-FAM (ET470/40x, ET525/50m) and the TxRed configuration for FM4-64 (ET560/40x, ET630/75m). The setup was outfitted with an environmental chamber that was kept at 30°C. Images were recorded with Andor Zyla 4.2 Plus sCMOS camera and images were captured with Nikon Elements (version 4.30) acquisition software. The following additional parameters were used: no binning, readout rate of 200 MHz, bit depth of 16-bit & dual gain ¼, sensor mode normal. Autoexposure for phase was between 200-400 ms, Bd-A-FAM samples were between 700 and 900 ms and for FM4-64 were between 200-400 ms. Autoexposure varied due to the slight variability of fluorophore concentrations and agar pad thicknesses between runs, but the same setup was used for all samples taken at a given time point. Imaging was performed at the MicRoN facility at Harvard Medical School (Boston, MA). Image adjustments for brightness and contrast were performed in ImageJ and are comparable between samples in the same time point (e.g. treated and untreated, WT and mutant, at 1 hour).

Transmission electron microscopy

*B. vulgatus* ATCC 8482 was inoculated and grown in basal for several hours until an OD_600_ of 0.25. At this time, Bd-A was added to a final concentration of 2 µg/mL. For the control, samples were grown with water added. The experimental and control cultures were grown for three hours, at which point 1 mL of each culture was collected and pelleted. Supernatant was removed and samples were resusupended in 1 mL of 1% glutaraldehyde in 0.1M cacodylate buffer and held overnight at 4$^{\circ}$C. The samples were prepared and imaged by the Harvard Medical School Electron Microscopy Facility (Boston, MA) using the following provided protocol: The pellets of cells were washed in 0.1M cacodylate buffer and postfixed with 1% osmiumtetroxide (OsO4)/1.5% potassium ferrocyanideK4[Fe(CN)6] for 1 hour, washed 2x in water, 1x Maleate buffer (MB) 1x and incubated in 1% uranyl acetate in MB for 1hr followed by 2 washes in water and subsequent dehydration in grades of alcohol (10min each; 50%, 70%, 90%, 2x10min 100%). The samples were then put in propyleneoxide for 1 hr and infiltrated overnight in a 1:1 mixture of propyleneoxide and Spurr’s low viscosity resin (Electron Microscopy sciences, Hatfield, PA). The following day the samples were embedded in Spurr’s resin and polymerized at 60 degrees C for 48 hrs. Ultrathin sections (about 80nm) were cut on a Reichert Ultracut-S microtome, picked up on to copper grids stained with lead citrate and examined in a JEOL 1200EX Transmission electron microscope images were recorded with an AMT 2k CCD camera.

RNAseq analysis

*B. vulgatus* ATCC 8482 was inoculated and grown in 10 mL of basal for several hours until an OD_600_ of 0.25. At this time, the culture was divided into two samples of 5 mL each. To one sample, Bd-A was added to a final concentration of 2 µg/mL. Both the treated and untreated cultures were grown until they reached an OD_600_ of 0.8, and 1 mL of each culture was collected and pelleted. This was repeated to obtain three biological replicates of treated and control samples. Pellets were sent for RNA sequencing at Novogene (South Plainfield, NJ). The Illumina reads were adapter- and quality-trimmed using utilities included in the BBMap package of bioinformatics tools (v. 38.90) and evaluated before and after trimming using the FastQC tool (v. 0.11.9). The reads were mapped to the *Bacteroides vulgatus* ATCC 8482 genome (NCBI accession NC_009614.1) using the Bowtie 2 short read aligner (v. 2.4.2, (3)). The SAM files output by Bowtie 2 were converted to sorted and indexed BAM files using SAMtools (v. 1.11, (4)), and compared to a General Feature Format (GFF) file of the *Bacteroides vulgatus* ATCC 8482 coding domain (CDS) intervals using BEDtools (v. 2.30.0, (5)). The read mapping results were evaluated for differential gene expression using both DESeq2 (v. 1.30.0, (6)) and edgeR (v. 3.32.1, (7)) as Bioconductor (v. 3.12, (8)) packages under R (v. 4.0.4) and RStudio (v. 1.2.5042). A gene was considered differentially expressed if the absolute value of the fold change of the expression level in the experimental samples compared to the control samples was greater than or equal to 2 and the adjusted p-value (padj for DESeq2 and FDR for edgeR) was less than or equal to 0.05, as calculated by both statistical packages. In cases where DESeq2 returned the designation “NA” due to Cook’s distance read count outliers or zero read count rows, edgeR calculations were relied on exclusively for determination of DEG.

COG and SusCD assignments and volcano plots

The CDD database files (v. 3.19, (9)) were downloaded from the NCBI FTP site and the position-specific scoring matrix (PSSM) files corresponding to COGs ((10)) were extracted and used to create a reverse-position specific blast database using the makeprofiledb program from the NCBI BLAST suite (v. 2.11.0, (11)). The amino acid sequences of all protein-encoding genes of the *Bacteroides vulgatus* ATCC 8482 genome were used as queries against this database using the rpsblast program (11). The output was parsed, and results exceeding the minimum bitscore threshold recorded in the bitscore_specific_3.19.txt file provided by NCBI were retained. The COGs remaining were clustered by group and category, and these clusters were used to generate volcano plots using GraphPad Prism version 9.1.1 for Windows (GraphPad Software, San Diego, California USA). SusCD genes were detected by use of five profile HMM files: PF07980.11 (SusD_RagB), PF12741.7 (SusD-like), PF12771.7 (SusD-like_2), and PF14322.6 (SusD-like_2) from the Sanger Pfam database (v. 34.0) and TIGR04057 (SusC_RagA_signa) from the TIGRfam database (v. 15.0). These models were used to create a database and searched with tools from HMMER (v. 3.3 (12)) using the calculated profile gathering threshold value as the cut off for significance.

Isolation and sequencing of BtCL15 strains

Human fecal sample collection was approved by the Mass General Brigham Human Research Committee IRB and complied with all relevant federal guidelines and institutional policies. The fecal sample collected at month 119 (T119) compared to the early fecal sample collected at month 12, was suspended in sterile PBS, then serially diluted and plated on LKV plates (Remel R01044). Single colonies were picked and recloned. Strains were tested by PCR for presence of the Bd-B locus and for production of Bd-B. BtCL15T12C11 and BtCL15T119C47 were subject to WGS. Cultures were grown anaerobically in basal medium to an OD600 of ~0.8 and genomic DNA was extracted using a CTAB/NaCl DNA extraction protocol followed by sodium acetate/ethanol precipitation. After DNA was collected, samples were sent for whole genome sequencing using PacBio Sequel v3 SMRTcell technology at the Genomics Resource Center from the University of Maryland. The genomes were assembled separately using Falcon/Unzip 1.2.0 (13) and Flye 2.8.2 (14) then reconciled using the Flye ‘subassemblies’ option. Consensus assemblies were polished using GCpp 2.0.0 (Pacific Biosciences). Prodigal 2.6.3 (15) was used for gene calling and annotation was performed using a customized version of Prokka 1.14.6 (16). Plasmid assignment of small contigs was based on circularization during assembly and using PlasFlow (17).

Arbitrarily primed PCR (AP-PCR) analysis

16S rRNA gene sequences were obtained by PCR from colonies isolated from the CL15 community at T0 and T119. Samples were sent for Sanger sequencing at Harvard Biopolymers Facility (Boston, MA). Species isolated at T0 and at T119 that had the same 16S rRNA gene sequences were strain typed by AP-PCR. gDNA of these samples were isolated using NEB Genomic DNA Isolation kit and concentration was assessed using Qubit dsDNA BR Assay Kit (Invitrogen). Each PCR had a volume of 20 µL and PCR was performed using TAQ Polymerase 2X Master Mix with 0.5 µM AP-PCR primer and 1 ng of gDNA. Three individual AP-PCRs were performed per strain, one reaction per AP-PCR primer. Reaction conditions for all three AP-PCRs were as previously described (18).

**References**

1. Kelley LA, Mezulis S, Yates CM, Wass MN, Sternberg MJE. 2015. The Phyre2 web portal for protein modeling, prediction and analysis. Nature Protocols 10:845-858.

2. Noinaj N, Kuszak AJ, Gumbart JC, Lukacik P, Chang H, Easley NC, Lithgow T, Buchanan SK. 2013. Structural insight into the biogenesis of beta-barrel membrane proteins. Nature 501:385-90.

3. Langmead B, Salzberg SL. 2012. Fast gapped-read alignment with Bowtie 2. Nat Methods 9:357-9.

4. Danecek P, Bonfield JK, Liddle J, Marshall J, Ohan V, Pollard MO, Whitwham A, Keane T, McCarthy SA, Davies RM, Li H. 2021. Twelve years of SAMtools and BCFtools. Gigascience 10.

5. Quinlan AR, Hall IM. 2010. BEDTools: a flexible suite of utilities for comparing genomic features. Bioinformatics 26:841-2.

6. Love MI, Huber W, Anders S. 2014. Moderated estimation of fold change and dispersion for RNA-seq data with DESeq2. Genome Biol 15:550.

7. Robinson MD, McCarthy DJ, Smyth GK. 2010. edgeR: a Bioconductor package for differential expression analysis of digital gene expression data. Bioinformatics 26:139-40.

8. Huber W, Carey VJ, Gentleman R, Anders S, Carlson M, Carvalho BS, Bravo HC, Davis S, Gatto L, Girke T, Gottardo R, Hahne F, Hansen KD, Irizarry RA, Lawrence M, Love MI, MacDonald J, Obenchain V, Oles AK, Pages H, Reyes A, Shannon P, Smyth GK, Tenenbaum D, Waldron L, Morgan M. 2015. Orchestrating high-throughput genomic analysis with Bioconductor. Nat Methods 12:115-21.

9. Lu S, Wang J, Chitsaz F, Derbyshire MK, Geer RC, Gonzales NR, Gwadz M, Hurwitz DI, Marchler GH, Song JS, Thanki N, Yamashita RA, Yang M, Zhang D, Zheng C, Lanczycki CJ, Marchler-Bauer A. 2020. CDD/SPARCLE: the conserved domain database in 2020. Nucleic Acids Res 48:D265-D268.

10. Tatusov RL, Fedorova ND, Jackson JD, Jacobs AR, Kiryutin B, Koonin EV, Krylov DM, Mazumder R, Mekhedov SL, Nikolskaya AN, Rao BS, Smirnov S, Sverdlov AV, Vasudevan S, Wolf YI, Yin JJ, Natale DA. 2003. The COG database: an updated version includes eukaryotes. BMC Bioinformatics 4:41.

11. Camacho C, Coulouris G, Avagyan V, Ma N, Papadopoulos J, Bealer K, Madden TL. 2009. BLAST+: architecture and applications. BMC Bioinformatics 10:421.

12. Eddy SR. 2011. Accelerated Profile HMM Searches. PLoS Comput Biol 7:e1002195.

13. Chin CS, Peluso P, Sedlazeck FJ, Nattestad M, Concepcion GT, Clum A, Dunn C, O'Malley R, Figueroa-Balderas R, Morales-Cruz A, Cramer GR, Delledonne M, Luo C, Ecker JR, Cantu D, Rank DR, Schatz MC. 2016. Phased diploid genome assembly with single-molecule real-time sequencing. Nat Methods 13:1050-1054.

14. Kolmogorov M, Yuan J, Lin Y, Pevzner PA. 2019. Assembly of long, error-prone reads using repeat graphs. Nat Biotechnol 37:540-546.

15. Hyatt D, Chen GL, Locascio PF, Land ML, Larimer FW, Hauser LJ. 2010. Prodigal: prokaryotic gene recognition and translation initiation site identification. BMC Bioinformatics 11:119.

16. Seemann T. 2014. Prokka: rapid prokaryotic genome annotation. Bioinformatics 30:2068-9.

17. Krawczyk PS, Lipinski L, Dziembowski A. 2018. PlasFlow: predicting plasmid sequences in metagenomic data using genome signatures. Nucleic Acids Res 46:e35.

18. Zitomersky NL, Coyne MJ, Comstock LE. 2011. Longitudinal analysis of the prevalence, maintenance, and IgA response to species of the order Bacteroidales in the human gut. Infect Immun 79:2012-20.
